# Fear at the time of the COVID-19 pandemic: validation of the Arabic version of the Four-Dimensional Symptom Questionnaire among Saudi-based respondents

**DOI:** 10.1192/bjo.2020.166

**Published:** 2021-01-12

**Authors:** Abdulaziz I Aljemaiah, Mugtaba Osman, Sarah Alharbi, Randa Alshehri, Esraa Mansoer Aldggag, Alaa Talal Aljoudi, Dina Smail Abdulsomad, Mohammed Abdulghani, Fawaz Alotaibi

**Affiliations:** Armed Forces Center for Psychiatric Care, Taif, Saudi Arabia; Armed Forces Center for Psychiatric Care, Taif, Saudi Arabia; Armed Forces Center for Psychiatric Care, Taif, Saudi Arabia; Armed Forces Center for Psychiatric Care, Taif, Saudi Arabia; Armed Forces Center for Psychiatric Care, Taif, Saudi Arabia; Armed Forces Center for Psychiatric Care, Taif, Saudi Arabia; Armed Forces Center for Psychiatric Care, Taif, Saudi Arabia; Armed Forces Center for Psychiatric Care, Taif, Saudi Arabia; and Department of Psychiatry, Faculty of Medicine, Zagazig University, Egypt; Armed Forces Center for Psychiatric Care, Taif, Saudi Arabia

**Keywords:** 4DSQ test, Arabic, COVID-19, validation, depression, anxiety

## Abstract

**Background:**

The COVID-19 pandemic has caused unprecedented stress and fear throughout the world.

**Aims:**

To evaluate the psychological effects of the COVID-19 pandemic on the Saudi public, and to examine the performance of the Arabic version of the Four-Dimensional Symptom Questionnaire (4DSQ) scale.

**Method:**

We conducted an online questionnaire-based cross-sectional survey of a sample of the Saudi public.

**Results:**

The study included 347 participants, who reported significantly higher levels of distress, depressive symptoms, anxiety symptoms and somatisation compared with a normative sample. Females scored higher in terms of somatisation, depression and anxiety symptoms, and distress. Obtaining COVID-19 information from friends and relatives was associated with higher levels of somatisation, depression and anxiety symptoms, and distress. Cronbach's alpha was 0.93 for the distress scale, 0.88 for the depression scale, 0.88 for the anxiety scale and 0.86 for the somatisation scale.

**Conclusions:**

Levels of psychological distress were high among the Saudi public during the COVID-19 pandemic. We found high reliability for the Arabic version of the 4DSQ scale. However, three items did not conform to the four-factor structure, namely, item 1: ‘During the past week, did you suffer from dizziness or feeling light-headed?’, item 20: ‘During the past week, did you suffer from disturbed sleep?’ and item 46: ‘During the past week did you ever think *I wish I was dead*?’.

The COVID-19 pandemic triggered substantial stress, fear and uncertainty all over the world.^1^ Deaths exceeded 1.016 million worldwide, and there were more than 34.2 million confirmed cases by 2 October 2020.^2^ The pandemic resulted in substantial psychological effects, with moderate to severe anxiety and depressive symptoms in over half of the population.^3^ In addition, sleep disruption was reported during the initial phase of the lockdown as people's quality of sleep was severely reduced.^4^ Many people followed physical distancing rules during the lockdown period and believed in their effect of curbing the virus spread.^5^ That had profound effects on travel plans and work schedules, with subsequent mood deterioration, anxiety and fearfulness.

There is a pressing need to quantify the psychological repercussions of COVID-19, particularly in Middle Eastern countries. The Four-Dimensional Symptom Questionnaire (4DSQ) measures the extent of somatisation, distress, anxiety and depression using established psychometric properties across many cultures and languages.^6,7^ The 4DSQ Arabic version was developed in 2016 by a group of Egyptian researchers through a procedure of translation of the English version into Arabic and back-translation into English;^8^ however, no validation study was carried out to examine its reliability and/or factor structure. This study was conducted to achieve two aims. First, to validate the Arabic version of 4DSQ in a Saudi sample during the COVID-19 pandemic. Second, to quantify the epidemiological features of the psychological effects of the COVID-19 pandemic in Saudi Arabia.

## Method

### Study design

This was a cross-sectional questionnaire-based descriptive study. The study included a self-selected sample of *n* = 347 respondents, 61.4% male and 38.6% female, with a median age of 35 years, from Taif, Saudi Arabia. The survey was conducted between April and May 2020, the period during which a state-wide curfew and lockdown was imposed, 24 h a day, 7 days a week, to curb the spread of coronavirus. Violators of curfew were subjected to substantial on-the-spot fines. In addition, all air and sea travel to and from the Kingdom of Saudi Arabia was suspended. The Arabic adaptation of the 4DSQ questionnaire was obtained with permission and posted online with an invitation to complete the questionnaire. The link was sent through social media. The Arabic 4DSQ questions were published on an online survey website with a cover letter explaining the purpose of the study and the expected time to complete the survey. An invitation to complete the survey was designed and sent out to all authors involved in the study. The link to the online questionnaire was made available through major social media outlets throughout the study period. The target population was all adult citizens based in Taif.

### The 4DSQ tool

The 4DSQ tool, freely available at www.4dsq.eu, has 50 items related to feelings in the past week.^9^ The 4DSQ questionnaire investigates four dimensions, with 16 questions each for distress and somatisation, 12 questions for anxiety and six questions for depression. The 4DSQ uses a time-frame reference of 7 days. Each item has a five-point scale response, with options of ‘no’ (zero points), ‘sometimes’ (one point), and ‘regularly’, ‘often’ and ‘very often or constantly’ (two points each). The Arabic version of the 4DSQ was developed later in 2016, but no psychometric analysis was performed. Permission was granted kindly by the main author of the 4DSQ, Dr B. Terluin, to use the Arabic version in our current investigation. The 4DSQ was readily translated from the Dutch language into Arabic under supervision of the original developer of 4SDQ by a group of Egyptian academics. The Arabic version is available freely from www.4dsq.eu. We copied the Arabic version directly from the website, with the author's written permission; we were not involved in its exact wording.

### Setting

The study was conducted on a sample of respondents from Taif, Saudi Arabia.

### Data analysis

Data were analysed using the R statistical software, version 3.4.1. Multiple generalised linear regression methods were used to estimate the effects of demographic factors on the scores and subscores of the 4DSQ. We included all demographic variables simultaneously in a full regression model with fixed effects:
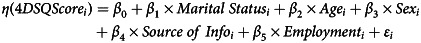


where the index *i* (ranging from 1 to 347) represents the data pertaining to individual *I*; ***β_0_–β_5_*** are estimates of the demographic effects; and η(.) is the linear predictor function for the Poisson distribution.

We considered each individual as an independent observation and used no random effects. We compared linear regression and Poisson regression models by examining the behaviour of the residuals in terms of normality and scatter when plotted against their corresponding fitted values. Poisson regression was superior in terms of model fit and diagnostics.

To compare the test results with the normative values, as shown in Table 2 below, we used the χ^2^-test to calculate *P*-values. We compared the counts observed in our sample with the normative population counts of the highest score class of factors, and related the association to the χ^2^ table with one degree of freedom.

We used the package ‘lavaan’ in R 3.4.1 to evaluate the latent factor structure of the 4DSQ subscales via confirmatory factor analyses.^10^ All the responses to the 50 4DSQ items were included as ordinal data in the model. To estimate factor loadings more accurately, we opted to use maximum likelihood with robust correction rather than diagonally weighted least squares.

### Ethical approval

The authors assert that all procedures contributing to this work comply with the ethical standards of the relevant national and institutional committees on human experimentation and with the Helsinki Declaration of 1975, as revised in 2008. All procedures involving human subjects or patients were approved by the Research and Ethics Committee Western Region, affiliated to the Medical Services General Directorate. Registration number: H-02-T-078.

### Consent

Written informed consent was obtained online from all participants.

## Results

The study took place between April 2020 and May 2020 in a self-selected sample of the public from Taif, Saudi Arabia.

The total number of respondents included in the study was *n* = 347 Saudi subjects. Only one (0.29%) individual returned the questionnaire incomplete, and these missing responses were excluded from the analysis.

There were *n* = 213 (61.4%) males and *n* = 134 (38.6%) females. The mean age was 35.5 years (s.d. = 10.3 years). The age range was 12 to 63 years. The median age was 35 years (i.e. 50% of the sample were older than 35 years). Table 1 presents in detail the basic demographic factors of the participants. The ‘information source’ is the reported method used most often by the respondents to obtain information about COVID-19, whether word of mouth from friends and relatives, state-based media news channels, official resources released by the Ministry of Health, or social media outlets.

**Table 1:** Baseline demographics of the study participants

Factor	Count (*n*)/mean	Percentage/s.d.
*Gender*		
Male	213	61.4%
Female	134	38.6%
Age	35.5 years	10.3 years
*Marital status*		
Married	228	65.7%
Widow	4	1.2%
Single	106	30.5%
Separated	9	2.6%
*Education*		
Diploma	24	6.9%
Primary	1	0.3%
Intermediate	13	3.7%
Secondary	52	15%
University	200	57.6%
Higher education	57	16.4%
*Employment*		
Health professional	56	16.1%
Public servant	122	35.2%
Military personnel	65	18.7%
Unemployed	18	5.2%
Student	18	5.2%
Retired	15	4.3%
Other	43	12.4%
Freelancer	5	1.4%
Housewife	5	1.4%
*Information source*		
Ministry of health	219	63.1%
Social media	59	17%
State media	62	17.9%
Friends and relatives	7	2%

Among the respondents, *n* = 32 (9.2%) scored over 20 in terms of distress, indicating ‘strongly elevated’ distress. Moreover, *n* = 43 (12.4%) had strongly elevated depression scores, and *n* = 22 (6.3%) reported strongly elevated anxiety. In addition, *n* = 11 (3.2%) had strongly elevated somatisation scores. These results are shown in Table 2. All indices were significantly higher than the corresponding normative values for the 4SDQ scale reported by Terluin et al.^11^ This is indicative of increased anxiety, depression, somatisation and distress during the COVID-19 pandemic among the Saudi public.

**Table 2:** 4DSQ results among participants

4DSQ scale	Count (%)	Count of normative data^11^ (%)	Chi-squared (2 d.f.)	*P*-value
Distress				
0–10	230 (66.3%)	4352 (82.5%)	56.826	<0.0001
11–20	85 (24.5%)	674 (12.8%)		
21–32	32 (9.2%)	249 (4.7%)		
Depression				
0–2	243 (70%)	4778 (90.6%)	144.39	<0.0001
3–5	61 (17.6%)	281 (5.3%)		
6–12	43 (12.4%)	216 (4.1%)		
Anxiety				
0–3	220 (63.4%)	4763 (90.3%)	246.1	<0.0001
4–9	105 (30.3%)	378 (7.2%)		
10–24	22 (6.3%)	132 (2.5%)		
Somatisation				
0–10	282 (81.3%)	4623 (87.7%)	13.904	0.0010
11–20	54 (15.6%)	572 (10.8%)		
21–32	11 (3.2%)	78 (1.5%)		

### Reliability and internal consistency of the Arabic 4DSQ

The reliability coefficient for the distress subscale of the Arabic 4DSQ was 0.93 (95% CI: 0.92–0.94), indicative of excellent internal consistency. None of its items had a significant effect on the overall reliability estimate. Similarly, the reliability coefficient for the depression subscale was 0.88 (95% CI: 0.85–0.89), and that for the anxiety subscale was 0.89 (95% CI: 0.87–0.90), indicative of good internal consistency in both cases. All of the items had comparable effects on the overall reliability estimate. Moreover, the reliability coefficient for the somatisation subscale was 0.86 (95% CI: 0.84–0.88), again indicative of good internal consistency. All its items had comparable effects on the overall reliability estimate. Further information is available in Supplementary File 1 available at https://10.1192/bjo.2020.166.

### Confirmatory factor analysis

We carried out a confirmatory factor analysis to ensure that the four dimensions were captured satisfactorily by the 4DSQ. However, the four-factor model did not fit in a satisfactory way for the full 50-item data-set. An overall five-factor structure was identified as the best compared with other models with lower numbers of factors. An additional dimension was required for the sample data to conform acceptably to the theoretical covariance structure. This was composed of item 1: ‘During the past week, did you suffer from dizziness or feeling light-headed?’, which was cross-loaded into the depression dimension; item 20: ‘During the past week, did you suffer from disturbed sleep?’, which was cross-loaded into the somatisation, depression and distress dimensions; and item 46: ‘During the past week, did you ever think *I wish I was dead*?’ which loaded on the additional *fifth* dimension only. See Supplementary File 3 for a detailed account of the five-factor loading.

We attempted to perform confirmatory factor analysis of a four-factor model after the removal of the three items above. The model was better than the four-factor model in the full data-set and of comparable acceptable fit to the five-factor model; see Table 3.

**Table 3:** Comparison of four models for the 4DSQ factor structure

Model	χ^2^ (df)	RMSEA (90% CI)	CFI	TLI	SRMR
One-factor	5526.536(1175)	0.104 (0.101 to 0.106)	0.959	0.957	0.135
Two-factor	5439.743 (1174)	0.102 (0.1 to 0.105)	0.960	0.958	0.117
Three-factor	5184 (1172)	0.099 (0.097 to 0.102)	0.962	0.960	0.132
Four-factor	3977.334 (1169)	0.083 (0.08 to 0.086)	0.973	0.972	0.116
Five-factor	3567.288 (1165)	0.077 (0.074 to 0.080)	0.977	0.976	0.112
Four-factor (excluding items 1, 20 & 46)	3566 (1165)	0.077 (0.074 to 0.081)	0.977	0.976	0.111

CFI, comparative fit index; TLI, Tucker–Lewis index; RMSEA, root mean square error of approximation; SRMR, standardised root mean square residual.

For a good fit, it is preferred that the comparative fit index and Tucker–Lewis index are greater than 0.95, and that the root mean square error of approximation (RMSEA) is less than 0.06.^12^ An RMSEA value less than 0.05 indicates a good fit to the data-set, and RMSEA less than 0.08 indicates an acceptable fit.^13^

### Effects of demographic factors on 4SDQ scores

#### Total 4DSQ scores

**Fig. 1:**
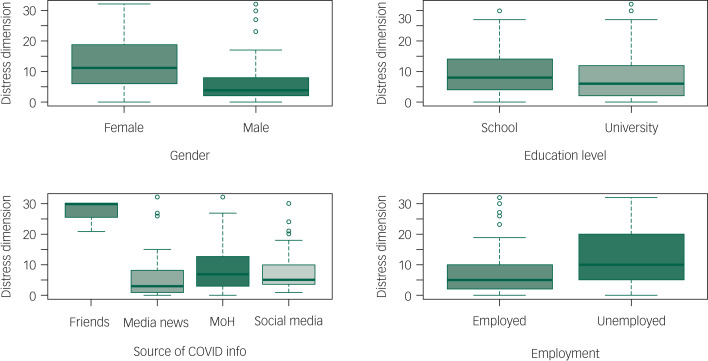
Effects of background factors on distress score.

**Fig. 2:**
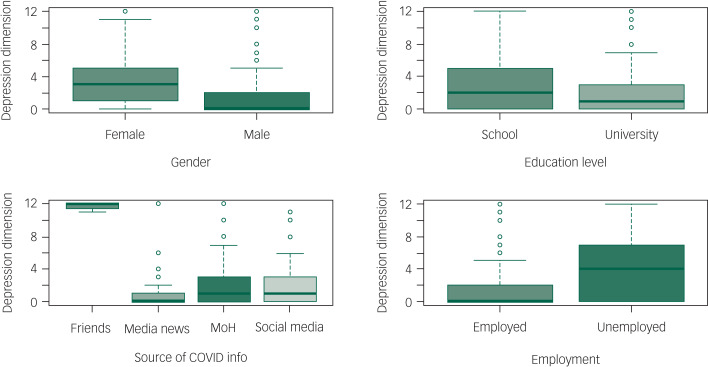
Effects of background factors on depression score.

**Fig. 3:**
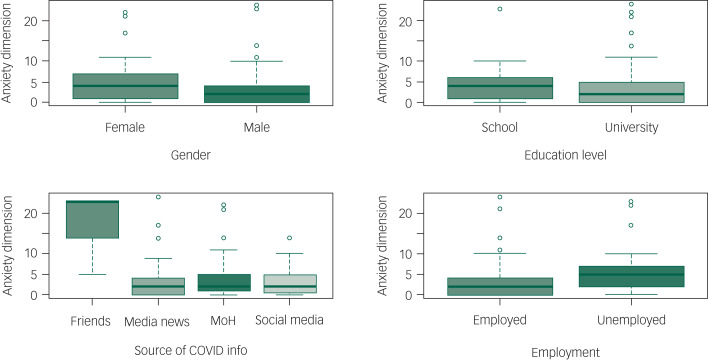
Effects of background factors on anxiety score.

**Fig. 4:**
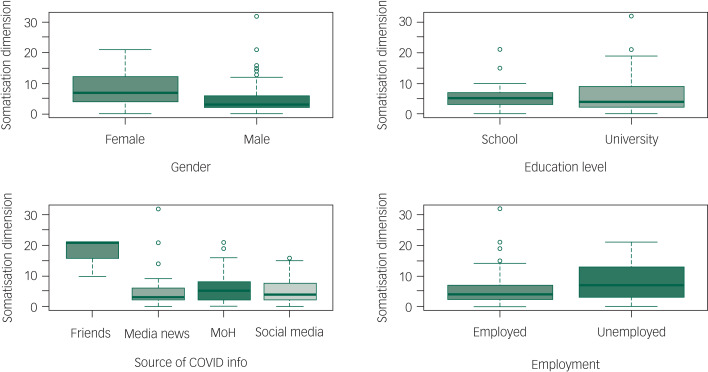
Effects of background factors on somatization score.

**Fig. 5:**
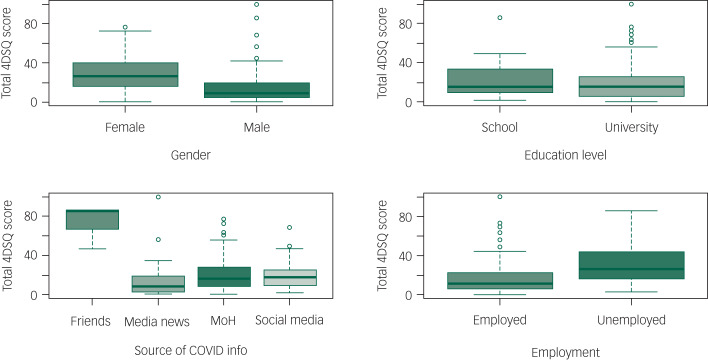
Effects of background factors on total 4DSQ score.

The total 4DSQ score was significantly increased in separated (incidence rate ratio [IRR] = 1.194 [95% CI: 1.013–1.396], *P* = 0.03), single (IRR = 1.08 [95% CI: 1.006–1.163], *P* = 0.034) and widowed (IRR = 1.416 [95% CI: 1.112–1.778], *P* = 0.004) individuals compared with those who were married. It was also increased by getting information from friends and relatives (IRR = 2.535 [95% CI: 2.281–2.811], *P* < 0.0001) and being unemployed (IRR = 1.127 [95% CI: 1.054–1.205], *P* < 0.0001). It was substantially reduced in participants who were older (each additional year of age decreased the IRR by 0.975 [95% CI: 0.972–0.978], *P* < 0.001), male (IRR = 0.705 [95% CI: 0.664–0.748], *P* < 0.0001), or university educated (IRR = 0.882 [95% CI: 0.828–0.941], *P* < 0.0001), and in those who used state media (IRR = 0.800 [95% CI: 0.743–0.861], *P* < 0.0001) or social media (IRR = 0.874 [95% CI: 0.816–0.935], *P* < 0.001) to get information related to COVID-19. See Table 4 , Figs. 1–5 and Supplementary File 2 for full details of the estimates of the effects of demographic variables on total and individual dimension 4DSQ scores.

**Table 4:** Estimates of the coefficients for effects of background factors on 4DSQ score and dimensions

	Estimate	IRR	95% CI	SE	*t*-test	*P*-value
Effect on total 4SDQ score						
Separated	0.177	1.194	1.013 to 1.396	0.082	2.165	0.030
Single	0.079	1.082	1.006 to 1.163	0.037	2.124	0.034
Widowed	0.348	1.416	1.112 to 1.778	0.120	2.909	0.004
Age	−0.025	0.975	0.972 to 0.978	0.002	−14.608	<0.001
Male	−0.350	0.705	0.664 to 0.748	0.030	−11.495	<0.001
University Edu	−0.125	0.882	0.828 to 0.941	0.033	−3.844	<0.001
Friends’ Info	0.930	2.535	2.281 to 2.811	0.053	17.451	<0.001
Media news	−0.223	0.800	0.743 to 0.861	0.038	−5.901	<0.001
Social media	−0.135	0.874	0.816 to 0.935	0.035	−3.898	<0.001
Unemployment	0.120	1.127	1.054 to 1.205	0.034	3.517	<0.001
Effect on distress dimension score						
Separated	0.378	1.459	1.148 to 1.830	0.119	3.186	0.001
Single	0.108	1.114	0.998 to 1.244	0.056	1.925	0.054
Widowed	0.461	1.586	1.101 to 2.213	0.178	2.595	0.009
Age	−0.031	0.969	0.965 to 0.975	0.003	−11.322	<0.001
Male	−0.355	0.701	0.639 to 0.769	0.047	−7.563	<0.001
University Edu	−0.177	0.838	0.759 to 0.925	0.050	−3.531	<0.001
Friends’ Info	0.743	2.102	1.772 to 2.483	0.086	8.643	<0.001
Media news	−0.288	0.750	0.666 to 0.841	0.060	−4.842	<0.001
Social media	−0.175	0.839	0.756 to 0.931	0.053	−3.289	0.001
Unemployment	0.032	1.033	0.932 to 1.143	0.052	0.611	0.541
Effect on depression dimension score						
Separated	0.081	1.084	0.661 to 1.683	0.237	0.343	0.731
Single	0.107	1.113	0.887 to 1.398	0.116	0.925	0.355
Widowed	0.505	1.657	0.857 to 2.918	0.310	1.629	0.103
Age	−0.017	0.983	0.974 to 0.993	0.005	−3.424	0.001
Male	−0.605	0.546	0.455 to 0.655	0.093	−6.518	<0.001
University Edu	−0.351	0.704	0.589 to 0.843	0.091	−3.838	<0.001
Friends’ Info	1.166	3.209	2.421 to 4.214	0.141	8.253	<0.001
Media news	−0.735	0.480	0.358 to 0.631	0.144	−5.085	<0.001
Social media	−0.104	0.901	0.737 to 1.096	0.101	−1.029	0.303
Unemployment	0.343	1.409	1.153 to 1.723	0.103	3.344	0.001
Effect on anxiety dimension score						
Separated	0.246	1.279	0.864 to 1.827	0.190	1.294	0.196
Single	−0.136	0.873	0.732 to 1.040	0.089	−1.524	0.128
Widowed	0.525	1.690	0.965 to 2.763	0.267	1.970	0.049
Age	−0.033	0.968	0.960 to 0.976	0.004	−7.899	<0.001
Male	−0.178	0.837	0.727 to 0.964	0.072	−2.471	0.013
University Edu	−0.231	0.794	0.685 to 0.921	0.076	−3.057	0.002
Friends’ Info	1.212	3.360	2.664 to 4.215	0.117	10.365	<0.001
Media news	−0.143	0.867	0.729 to 1.024	0.087	−1.653	0.098
Social media	−0.122	0.885	0.753 to 1.036	0.081	−1.500	0.133
Unemployment	0.133	1.142	0.970 to 1.346	0.083	1.600	0.110
Effect on somatisation dimension score						
Separated	−0.169	0.845	0.590 to 1.171	0.174	−0.967	0.333
Single	0.155	1.168	1.021 to 1.336	0.068	2.268	0.023
Widowed	−0.091	0.913	0.507 to 1.507	0.276	−0.330	0.741
Age	−0.017	0.983	0.977 to 0.990	0.003	−5.140	<0.001
Male	−0.351	0.704	0.630 to 0.787	0.057	−6.182	<0.001
University Edu	0.143	1.154	1.018 to 1.311	0.064	2.220	0.026
Friends’ Info	0.883	2.418	1.958 to 2.967	0.106	8.331	<0.001
Media news	−0.046	0.955	0.839 to 1.084	0.065	−0.703	0.482
Social media	−0.092	0.912	0.801 to 1.036	0.066	−1.398	0.162
Unemployment	0.154	1.166	1.031 to 1.321	0.063	2.439	0.015

#### Distress scores

The distress dimension score was significantly increased in separated (IRR = 1.459 [95% CI: 1.148–1.830], *P* = 0.001) and widowed individuals (IRR = 1.586 [95% CI: 1.101–2.213], *P* = 0.009) compared with those who were married. It was also increased by getting information from friends and relatives (IRR = 2.102 [95% CI: 1.772–2.483], *P* < 0.0001), but it was substantially reduced in participants who were older (each additional year of age decreased the IRR by 0.969 [95% CI: 0.965–0.975], *P* < 0.001), male (IRR = 0.701 [95% CI: 0.639–0.769], *P* < 0.001) or university educated (IRR = 0.838 [95% CI: 0.759–0.925], *P* < 0.0001), and in those who got information related to COVID-19 from state media (IRR = 0.750 [95% CI: 0.666–0.841], *P* < 0.001) or social media (IRR = 0.839 [95% CI: 0.756–0.931], *P* < 0.001).

Neither single marital status nor unemployment had a significant effect on the distress dimension score. See Table 4 and Supplementary File 2 for full details of the estimates of the effects of demographic variables on total and dimension 4DSQ scores.

#### Depression dimension score

The depression dimension score was significantly increased by getting information from friends and relatives (IRR = 3.209 [95% CI: 2.421–4.214], *P* < 0.001) and being unemployed (IRR = 1.409 [95% CI: 1.153–1.723], *P* = 0.001). It was substantially reduced in participants who were older (each additional year of age decreased IRR by 0.983 [95% CI: 0.974–0.993], *P* < 0.001), male (IRR = 0.546 [95% CI: 0.455–0.655], *P* < 0.0001) or university educated (IRR = 0.704 [95% CI: 0.589–0.843], *P* < 0.001), and in those who used state media (IRR = 0.480 [95% CI: 0.358–0.631], *P* < 0.001) to get information related to COVID-19.

Marital status and social media information did not affect depression dimension scores significantly. See Table 4 and Supplementary File 2 for full details of the estimates of the effects of demographic variables on total and dimension 4DSQ scores.

#### Anxiety dimension score

The anxiety dimension score was significantly increased by getting information from friends and relatives (IRR = 3.360 [95% CI: 2.664–4.215], *P* < 0.001) but substantially reduced in participants who were older (each additional year of age decreased IRR by 0.968 [95% CI: 0.960–0.976], *P* < 0.001), male (IRR = 0.837 [95% CI: 0.727–0.964], *P* = 0.013) or university educated (IRR = 0.794 [95% CI: 0.685–0.921], *P* = 0.002). Neither employment status nor use of state media or social media as a source of information significantly affected anxiety dimension scores. See Table 4 and Supplementary File 2 for full details of the estimates of the effects of demographic variables on total and dimension 4DSQ scores.

#### Somatisation dimension score

The somatisation dimension score was significantly increased by being single (IRR = 1.168 [95% CI: 1.021–1.336], *P* = 0.023), university educated (IRR = 1.154 [95% CI: 1.018–1.311], *P* = 0.026) or unemployed (IRR = 1.166 [95% CI: 1.031–1.321], *P* = 0.015) and by getting information from friends and relatives (IRR = 2.418 [95% CI: 1.958–2.967], *P* < 0.001). It was substantially reduced in older (each additional year of age decreased IRR by 0.983 [95% CI: 0.977 to 0.990], *P* < 0.001) and male (IRR = 0.704, *P* < 0.001) participants. Obtaining information from state or social media information did not significantly affect the somatisation dimension score. See Table 4 and Supplementary File 2 for full details of the estimates of the effects of demographic variables on total and dimension 4DSQ scores.

## Discussion

Current times are extremely tough as the COVID-19 pandemic is flaring everywhere in the world. We provided some evidence through the results of our survey of increased levels of distress, depression, anxiety and somatisation symptoms among the Saudi public during the time of the COVID-19 pandemic. This provides grounds for genuine concern regarding the pandemic-associated psychosocial effects in Saudi Arabia. Our findings are consistent with the results of a recent systematic review of the psychological effects of the COVID-19 pandemic, which estimated a prevalence of 33% for depression and 28% for anxiety.^14^ A recent Italian study found increased levels of distress among the public, which, in turn, influenced the perceived negative effects of the COVID-19 crisis.^15^ Many factors have affected distress levels among the public during the current crisis. Lockdown measures and curfews have negative effects on people, particularly those unable to sustain themselves with such measures in place, leading to increased levels of anxiety and depression.^16^ Worst, the evidence suggests that such high levels of distress, anxiety and poverty lead to a high risk of contracting COVID-19 and a higher risk of complications.^17^ It has been theorised that COVID-19 infection activates the same immune–inflammatory pathways that are associated with a range of mental health difficulties.^18^

One other factor that has been proposed to increase the psychological effects of COVID-19 is a lack of satisfaction with health information.^19^ Although social media may spread enormous amounts of misleading and anxiety-provoking material about COVID-19, some countries have managed to use social media effectively to promote accurate public health advice and hence to limit feelings of depression and anxiety among the public.^20^

One striking finding in our survey was the substantial negative effect of getting informal information from friends and relatives on all dimensions of psychological experiences. Word of mouth is quite influential in the cohesive tribal Saudi community, and many studies have found that it may form the main source of information about many medical and health-related issues.^21,22^ Information about COVID-19 evolves by the hour, given its novel mechanisms. Hence, harmful rumours tend to spread widely via informal channels of information.^23^

We identified females to be at increased risk of distress, depression and somatisation during the current COVID-19 crisis in Saudi Arabia. Many studies have confirmed that women are more susceptible to psychological distress during the current pandemic.^24^ Females were also more likely to suffer depressive symptoms, particularly among healthcare workers. Biological factors could explain such differences in emotional susceptibility between men and women.

Our results confirm the high reliability for the scores of the four dimensions of the Arabic version of the 4DSQ scale. This corroborates good psychometric properties for the 4DSQ in primary care facilities.^25^ Some authors went on to use the 4DSQ as a gold standard against which other psychometric tools could be compared.^26^ The Cronbach's alpha values for the four dimensions of the 4DSQ tool in its Arabic format indicated its high reliability and high internal consistency. Thus, the Arabic 4DSQ could be used reliably in various research and clinical settings to evaluate the presence and extent of symptoms of distress, depression, anxiety and somatisation among Arabic speakers.

We also demonstrated that for the Arabic 4DSQ to conform to the underlying four-factor structure, three items require removal. It is hard to explain why those three questions do not conform to the overall four-factor structure. One potential explanation is the repetition of their concepts in other items. For instance, item 20 ‘During the past week, did you suffer from disturbed sleep?’ and item 39 ‘During the past week, did you have difficulty in getting to sleep?’ seem to measure the same construct, although they belong to different dimensions. Moreover, death wishes are explored using both item 46 ‘During the past week, did you ever think *I wish I was dead*?’ and item 33 ‘During the past week, did you feel that you would be better off if you were dead?’. A clearer explanation could be found should the factor analysis be carried out in a large-scale face-to-face investigation. Of note, many studies have confirmed the factor structure in different languages, including Polish,^27^ Russian^28^ and Turkish.^29^ However, as we examined the Arabic version of 4DSQ, we concluded that three items need revision before it can effectively measure the underlying four constructs in Arabic-speaking populations.

The current investigation, to the best of our knowledge, was the first validation of the Arabic version of the 4DQS. We presented the results of a study with many strengths. We used an established and well-validated tool. Our sample included a wide range of participants that could represent the Saudi public at large. We used robust statistical methods to model our data-set. However, several limitations must be acknowledged. Our survey was cross-sectional and observational and thus would allow for limited generalisability. The online survey was subject to technological availability and computer literacy. It was, however, the only reasonable option during the time of the COVID-19 pandemic. Furthermore, although the questionnaire was online and was theoretically accessible to all Saudi citizens, the recruitment invitation was sent out through social media outlets that were primarily used by local Taif-based people. It would be more accurate to assume that the obtained sample is representative of local Taif residents than of all Saudi Arabia. Moreover, the high education level observed in our survey was not representative of the wider Saudi population. It can be attributed to our respondents being those who had access to social media and were more willing to complete the online survey. Similarly, the nature of the online survey may have left out older and retired respondents. The low participation by females could well be related to unique Saudi cultural, social and religious restrictions. However, substantial changes are underway in Saudi Arabia; hence, future surveys should suffer less from this gender difference in participation. Moreover, we did not use a completely matched control from Saudi Arabia for our normative data. The normative data we used were different in terms of geographical localisation, method of interview and recruitment practicality.

We need to be cautious before directly attributing detrimental effects on mental health to the COVID-19 pandemic or the curfew. We must consider the effects of limited access to mental health services during the pandemic.

Future research should include a large number of respondents (preferably thousands) and, as the pandemic eases, should use face-to-face measures. We did not collect data pertaining to the effects of ethnicity on 4DSQ scores. Future research should consider that in more depth.

Psychological sequelae to the COVID-19 pandemic are quite prevalent and should be addressed by public health measures hand in hand with medical sequelae. Furthermore, the provision of accurate information about COVID-19 by the state media and Ministry of Health is an important factor in alleviating psychological distress during the COVID-19 crisis. The Arabic version of the 4DSQ is reliable and valid; however, it requires reconsideration of three repetitive items before it can safely be used in primary and specialist care settings in Saudi Arabia and the Arabic-speaking world.

Based on our results, public health campaigns should clearly advise the public to avoid word-of-mouth information regarding the COVID-19 pandemic and coronavirus. The public should prefer institutional and official communications provided through the Ministry of Health, governmental sites and media outlets.

## Data Availability

The data that support the findings of this study are available from the corresponding author, M.O., upon reasonable request.

## Supplementary material

For supplementary material accompanying this paper visit https://doi.org/10.1192/bjo.2020.166. click here to view supplementary material
