# Supplementary material for: Fear at the time of the COVID-19 pandemic: validation of the Arabic version of the Four-Dimensional Symptom Questionnaire among Saudi-based respondents
Source: BJPsych Open. 2021 Jan 12;7(1):e33. doi: 10.1192/bjo.2020.166 (PMC7804080; doi:10.1192/bjo.2020.166)
Supplement: Supplementary file 1 [file S2056472420001660sup.zip › S2056472420001660sup001.docx]

Somatization

Supposed: A1+A2+A3+A4+A5+A6+A7+A8+A9+A10+A11+A12+A13+A14+A15+A16

Best: A2+ A4+ A5+A7+A8+A9+A10+A11+A12+A13+A14+A15+A16+A43

Let us examine:

A1 missing: dizziness or feeling light-headed?

A3 missing: fainting?

A6 missing: excessive sweating?

A43 added: were you afraid to travel on buses, streetcars/ trams, subways or trains?

Depression

Supposed: A28+A30+A33+A34+A35+A46

Best: A17+A22+A23+A27+A28+A29+A30+A31+A32+A33+A34+A35+A36+A37+A38+A40+A41+A42+A44+A47

Let us examine:

A46 missing: did you ever think "I wish I was dead"?

A17 added: feeling down or depressed?

A22 added: lack of energy?

A23 added: trembling when with other people?

A27 added: frightened?

A29 added: that you just can't do anything anymore?

A31 added: that you can no longer take any interest in the people and things around you?

A32 added: that you can't cope anymore?

A36 added: that you can't face it anymore?

A37 added: no longer feel like doing anything?

A38 added: have difficulty in thinking clearly?

A40 added: have any fear of going out of the house alone?

A44 added: were you afraid of becoming embarrassed when with other people?

A47 added: did you ever have fleeting images of any upsetting event(s) that you have experienced?

Anxiety

Supposed: A18+A21+A23+A24+A27+A40+A42+A43+A44+A45+A49+A50

Best: A6+A11+A18+A21+A24+A25+A45+A49+A50

Let us examine:

A23 missing: trembling when with other people?

A27 missing: frightened?

A40 missing: have any fear of going out of the house alone?

A42 missing: were you afraid of anything when there was really no need for you to be afraid?

A43 missing: were you afraid to travel on buses, streetcars/ trams, subways or trains?

A44 missing: were you afraid of becoming embarrassed when with other people?

A6 added: excessive sweating?

A11 added: shortness of breath?

A21 added: a vague feeling of fear?

A25 added: tense?

Distress

Supposed: A17+A19+A20+A22+A25+A26+A29+A31+A32+A36+A37+A38+A39+A41+A47+A48

Best: A1+A3+A19+A20+A26+A39+A46+A48

Let us examine:

A17 missing: feeling down or depressed?

A22 missing: lack of energy?

A25 missing: tense?

A29 missing: that you just can't do anything anymore?

A31 missing: that you can no longer take any interest in the people and things around you?

A32 missing: that you can't cope anymore?

A36 missing: that you can't face it anymore?

A37 missing: no longer feel like doing anything?

A38 missing: have difficulty in thinking clearly?

A41 missing: did you easily become emotional?

A47 missing: did you ever have fleeting images of any upsetting event(s) that you have experienced?

A1 added: dizziness or feeling light-headed

A3 added: fainting?

A46 added: did you ever think "I wish I was dead"?

STEP ONE RECONCILIATION: substantive changes each dimension

A17 “did you ever have fleeting images of any upsetting event(s) that you have experienced?” **from distress to depression**

A25 “tense” **from distress to anxiety**

A6 “excessive sweating?” **from somatization to anxiety**

A1 “ dizziness or feeling light-headed?” **from somatization to anxiety**

model.console.1 <- '

dis =~ A19+A20+A22 +A26+A29+A31+A32+A36+A37+A38+A39+A41+A47+A48

dep =~ A28+A30+A33+A34+A35+A46+ A17

anx =~ A1+A6+A18+A21+A23+A24+A27+A40+A42+A43+A44+A45+A49+A50+A25

som =~ A2+A3+A4+A5 +A7+A8+A9+A10+A11+A12+A13+A14+A15+A16

'

Result: No much improvement:

chisq df pvalue cfi rmsea bic srmr tli

4104.700 1169.000 0.000 0.972 0.085 NA 0.119 0.971

STEP TWO RECONCILIATION: depression to anxiety dimension

A17 “did you ever have fleeting images of any upsetting event(s) that you have experienced?” **from distress to depression**

A25 “tense” **from distress to anxiety**

A6 “excessive sweating?” **from somatization to anxiety**

A1 “ dizziness or feeling light-headed?” **from somatization to anxiety**

A41 “ did you easily become emotional?” **from distress to anxiety**

A22 “lack of energy?” **from distress to depression**

A29 “ that you just can't do anything anymore?” **from distress to depression**

A31 “ that you can no longer take any interest in the people and things around you?” **from distress to depression**

A37 “ no longer feel like doing anything?” **from distress to depression**

model.console.2 <- '

dis =~ A19+A20+A26+A32+A36+A38+A39 +A47+A48

dep =~ A28+A30+A33+A34+A35+A46+ A17 +A22 +A29+A31+A37

anx =~ A1+A6+A18+A21+A23+A24+A27+A40+A41+A42+A43+A44+A45+A49+A50+A25

som =~ A2+A3+A4+A5 +A7+A8+A9+A10+A11+A12+A13+A14+A15+A16

'

Result: No much improvement:

chisq df pvalue cfi rmsea bic srmr tli

4145.713 1169.000 0.000 0.972 0.086 NA 0.120 0.970

STEP THREE RECONCILIATION: vacating somatization dimension

A17 “did you ever have fleeting images of any upsetting event(s) that you have experienced?” **from distress to depression**

A25 “tense” **from distress to anxiety**

A6 “excessive sweating?” **from somatization to anxiety**

A1 “ dizziness or feeling light-headed?” **from somatization to anxiety**

A41 “ did you easily become emotional?” **from distress to anxiety**

A22 “lack of energy?” **from distress to depression**

A29 “ that you just can't do anything anymore?” **from distress to depression**

A31 “ that you can no longer take any interest in the people and things around you?” **from distress to depression**

A37 “ no longer feel like doing anything?” **from distress to depression**

A40 “ have any fear of going out of the house alone?” **from anxiety to depression**

A44 “ were you afraid of becoming embarrassed when with other people?” **from anxiety to depression**

model.console.3 <- '

dis =~ A19+A20+A26+A32+A36+A38+A39 +A47+A48

dep =~ A28+A30+A33+A34+A35+A46+ A17 +A22 +A29+A31+A37+A40+A44

anx =~ A1+A6+A18+A21+A23+A24+A27 +A41+A42+A43 +A45+A49+A50+A25

som =~ A2+A3+A4+A5 +A7+A8+A9+A10+A11+A12+A13+A14+A15+A16

'

Result: Minimal improvement:

chisq df pvalue cfi rmsea bic srmr tli

4078.503 1169.000 0.000 0.972 0.085 NA 0.119 0.971

Exploratory factor analysis with 5 factors

Factor1 Factor2 Factor3 Factor4 Factor5

dat.DS28 0.580

dat.DS30 0.753

dat.DS33 0.775 0.365

dat.DS34 0.766

dat.DS35 0.751

dat.DS40 0.583

dat.DS44 0.556

dat.DS29 0.762

dat.DS31 0.803

dat.DS32 0.727

dat.DS36 0.858

dat.DS37 0.705

dat.DS38 0.622

dat.DS47 0.610 0.377

dat.DS2 0.773

dat.DS4 0.530

dat.DS5 0.578

dat.DS13 0.546

dat.DS14 0.625

dat.DS18 0.635

dat.DS21 0.707

dat.DS24 0.568

dat.DS27 0.366 0.711

dat.DS45 0.439 0.571

dat.DS50 0.586

dat.DS25 0.534

dat.DS26 0.532

dat.DS46 0.516

dat.DS23 0.464 0.394

dat.DS42 0.460

dat.DS43 0.488

dat.DS49 0.416

dat.DS1 0.484 0.419

dat.DS3

dat.DS6 0.383

dat.DS7 0.386

dat.DS8 0.457

dat.DS9 0.384

dat.DS10 0.395

dat.DS11 0.368

dat.DS12 0.495

dat.DS15 0.459

dat.DS16 0.455

dat.DS17 0.491 0.429

dat.DS19 0.373 0.371 0.478

dat.DS20

dat.DS22 0.433 0.415 0.406

dat.DS39

dat.DS41 0.436

dat.DS48 0.388 0.387

Factor1 Factor2 Factor3 Factor4 Factor5

SS loadings 9.850 5.475 5.206 2.325 1.759

Proportion Var 0.197 0.109 0.104 0.046 0.035

Cumulative Var 0.197 0.306 0.411 0.457 0.492

Test of the hypothesis that 5 factors are sufficient.

The chi square statistic is 4211.06 on 985 degrees of freedom.

The p-value is 0

SUPERIOR FIT

chisq df pvalue cfi rmsea bic srmr tli

3567.288 1165.000 0.000 0.977 0.077 NA 0.112 0.976

Uniquenesses:

dat.DS28 dat.DS30 dat.DS33 dat.DS34 dat.DS35 dat.DS46 dat.DS18 dat.DS21 dat.DS23

0.434 0.337 0.224 0.295 0.368 0.436 0.437 0.342 0.549

dat.DS24 dat.DS27 dat.DS40 dat.DS42 dat.DS43 dat.DS44 dat.DS45 dat.DS49 dat.DS50

0.568 0.265 0.616 0.678 0.534 0.490 0.421 0.716 0.522

dat.DS1 dat.DS2 dat.DS3 dat.DS4 dat.DS5 dat.DS6 dat.DS7 dat.DS8 dat.DS9

0.494 0.345 0.820 0.711 0.609 0.746 0.683 0.707 0.724

dat.DS10 dat.DS11 dat.DS12 dat.DS13 dat.DS14 dat.DS15 dat.DS16 dat.DS17 dat.DS19

0.770 0.640 0.632 0.612 0.500 0.612 0.616 0.428 0.354

dat.DS20 dat.DS22 dat.DS25 dat.DS26 dat.DS29 dat.DS31 dat.DS32 dat.DS36 dat.DS37

0.734 0.456 0.475 0.452 0.327 0.228 0.319 0.188 0.346

dat.DS38 dat.DS39 dat.DS41 dat.DS47 dat.DS48

0.380 0.716 0.684 0.373 0.473

Loadings:

Factor1 Factor2 Factor3 Factor4 Factor5

dat.DS28 0.580 0.260 0.224 0.252 0.219

dat.DS30 0.753 0.214

dat.DS33 0.775 0.365

dat.DS34 0.766 0.270

dat.DS35 0.751 0.204

dat.DS40 0.583

dat.DS44 0.556 0.323 0.293

dat.DS29 0.762 0.224

dat.DS31 0.803 0.236

dat.DS32 0.727 0.246 0.215

dat.DS36 0.858 0.225

dat.DS37 0.705 0.310 0.209

dat.DS38 0.622 0.305 0.239 0.278

dat.DS47 0.610 0.331 0.377

dat.DS2 0.773

dat.DS4 0.530

dat.DS5 0.578 0.230

dat.DS13 0.546

dat.DS14 0.625 0.292

dat.DS18 0.262 0.239 0.635

dat.DS21 0.316 0.707 0.219

dat.DS24 0.283 0.568

dat.DS27 0.366 0.711 0.237

dat.DS45 0.439 0.571

dat.DS50 0.329 0.586

dat.DS25 0.287 0.204 0.534 0.318

dat.DS26 0.291 0.318 0.215 0.532

dat.DS46 0.258 0.229 0.309 0.287 0.516

dat.DS23 0.464 0.244 0.394

dat.DS42 0.460 0.306

dat.DS43 0.262 0.488 0.324

dat.DS49 0.289 0.416

dat.DS1 0.251 0.484 0.419

dat.DS3 0.240 0.336

dat.DS6 0.304 0.383

dat.DS7 0.232 0.386 0.261 0.208

dat.DS8 0.201 0.457

dat.DS9 0.384 0.276

dat.DS10 0.261 0.395

dat.DS11 0.203 0.297 0.368 0.295

dat.DS12 0.495 0.206 0.238

dat.DS15 0.216 0.459 0.288 0.218

dat.DS16 0.254 0.455 0.315

dat.DS17 0.491 0.239 0.216 0.429 0.208

dat.DS19 0.333 0.373 0.371 0.478

dat.DS20 0.210 0.283 0.309

dat.DS22 0.433 0.415 0.406

dat.DS39 0.261 0.284 0.210 0.301

dat.DS41 0.436 0.338

dat.DS48 0.388 0.237 0.282 0.387 0.303

Factor1 Factor2 Factor3 Factor4 Factor5

SS loadings 9.850 5.475 5.206 2.325 1.759

Proportion Var 0.197 0.109 0.104 0.046 0.035

Cumulative Var 0.197 0.306 0.411 0.457 0.492

Test of the hypothesis that 5 factors are sufficient.

The chi square statistic is 4211.06 on 985 degrees of freedom.
