# Supplementary material for: Fear at the time of the COVID-19 pandemic: validation of the Arabic version of the Four-Dimensional Symptom Questionnaire among Saudi-based respondents
Source: BJPsych Open. 2021 Jan 12;7(1):e33. doi: 10.1192/bjo.2020.166 (PMC7804080; doi:10.1192/bjo.2020.166)
Supplement: Supplementary file 1 [file S2056472420001660sup.zip › S2056472420001660sup002.docx]

**Table 1 Reliability coefficients for the 4DSQ subscales**

| **4DSQ scale** | **Reliability estimates** | | | | **SEM*** |
| --- | --- | --- | --- | --- | --- |
|  | **Cronbach’s alpha** | **Omega-total** | **Omega-hierarchical** | **Omega asymptotic** |  |
| **Distress** | 0.93 (0.92-0.94) | 0.95 | 0.79 | 0.83 | 2.057 |
| **Depression** | 0.88 (0.85-0.89) | 0.91 | 0.64 | 0.70 | 1.089 |
| **Anxiety** | 0.89 (0.87-0.90) | 0.91 | 0.75 | 0.83 | 1.613 |
| **Somatization** | 0.86 (0.84-0.88) | 0.89 | 0.58 | 0.66 | 1.942 |
| **4DSQ total** | 0.96 (0.95-0.96) | 0.97 | 0.66 | 0.68 | 3.614 |
| SEM Standard Errors of Measurement =  standard deviation * √(1 – reliability) | | | | | |

# Cronbach’s Alpha for Distress dimension

| raw α | std. α | G6(smc) | average r | S/N | ase | mean | sd | median_r |
| --- | --- | --- | --- | --- | --- | --- | --- | --- |
| 0.93 | 0.93 | 0.95 | 0.46 | 13 | 0.0055 | 0.53 | 0.49 | 0.45 |

- raw α: alpha based upon the covariances
- std. α: The standardized alpha based upon the correlations
- G6(smc): Guttman's Lambda 6 reliability
- smc = (the squared multiple correlation)
- Average r: The average interitem correlation
- S/N: Signal/Noise ratio (the ratio of reliable variance to unreliable variance)
- Ase: alpha standard error
- mean: For data matrices, the mean of the scale formed by summing the items
- sd: For data matrices, the standard deviation of the total score
- r: The correlation of each item with the total score (not corrected for item overlap)
- median-r: The median interitem correlation

| lower95% confidence boundary | alpha | upper 95% confidence boundary |
| --- | --- | --- |
| 0.92 | 0.93 | 0.94 |

Reliability if an item is dropped:

|  | raw_α | std.α | G6(smc) | average r | S/N | alpha se | var.r | med.r |
| --- | --- | --- | --- | --- | --- | --- | --- | --- |
| Item17 | 0.92 | 0.93 | 0.94 | 0.45 | 12 | 0.0060 | 0.017 | 0.44 |
| Item19 | 0.92 | 0.93 | 0.94 | 0.45 | 12 | 0.0060 | 0.018 | 0.44 |
| Item20 | 0.93 | 0.93 | 0.94 | 0.47 | 13 | 0.0055 | 0.014 | 0.47 |
| Item22 | 0.92 | 0.93 | 0.94 | 0.46 | 13 | 0.0060 | 0.018 | 0.45 |
| Item25 | 0.93 | 0.93 | 0.95 | 0.46 | 13 | 0.0058 | 0.018 | 0.46 |
| Item26 | 0.93 | 0.93 | 0.95 | 0.46 | 13 | 0.0059 | 0.018 | 0.45 |
| Item29 | 0.92 | 0.92 | 0.94 | 0.45 | 12 | 0.0060 | 0.016 | 0.45 |
| Item31 | 0.92 | 0.92 | 0.94 | 0.45 | 12 | 0.0061 | 0.015 | 0.45 |
| Item32 | 0.92 | 0.92 | 0.94 | 0.45 | 12 | 0.0060 | 0.016 | 0.45 |
| Item36 | 0.92 | 0.92 | 0.94 | 0.45 | 12 | 0.0060 | 0.015 | 0.45 |
| Item37 | 0.92 | 0.92 | 0.94 | 0.45 | 12 | 0.0061 | 0.016 | 0.45 |
| Item38 | 0.92 | 0.92 | 0.94 | 0.45 | 12 | 0.0061 | 0.016 | 0.44 |
| Item39 | 0.93 | 0.93 | 0.94 | 0.47 | 13 | 0.0056 | 0.016 | 0.47 |
| Item41 | 0.93 | 0.93 | 0.95 | 0.48 | 14 | 0.0055 | 0.015 | 0.48 |
| Item47 | 0.93 | 0.93 | 0.94 | 0.46 | 13 | 0.0058 | 0.017 | 0.45 |
| Item48 | 0.92 | 0.93 | 0.94 | 0.46 | 13 | 0.0059 | 0.018 | 0.45 |

Item statistics

|  | n | raw.r | std.r | r.cor | r.drop | mean | sd |
| --- | --- | --- | --- | --- | --- | --- | --- |
| Item17 | 347 | 0.75 | 0.75 | 0.73 | 0.70 | 0.58 | 0.69 |
| Item19 | 347 | 0.74 | 0.74 | 0.72 | 0.70 | 0.62 | 0.67 |
| Item20 | 347 | 0.56 | 0.55 | 0.53 | 0.49 | 0.70 | 0.75 |
| Item22 | 347 | 0.72 | 0.72 | 0.69 | 0.67 | 0.90 | 0.72 |
| Item25 | 347 | 0.64 | 0.65 | 0.62 | 0.59 | 0.44 | 0.64 |
| Item26 | 347 | 0.69 | 0.68 | 0.66 | 0.64 | 0.80 | 0.76 |
| Item29 | 347 | 0.75 | 0.76 | 0.75 | 0.71 | 0.33 | 0.67 |
| Item31 | 347 | 0.79 | 0.79 | 0.79 | 0.75 | 0.36 | 0.68 |
| Item32 | 347 | 0.76 | 0.77 | 0.76 | 0.72 | 0.36 | 0.66 |
| Item36 | 347 | 0.76 | 0.77 | 0.77 | 0.73 | 0.27 | 0.60 |
| Item37 | 347 | 0.79 | 0.79 | 0.78 | 0.74 | 0.51 | 0.74 |
| Item38 | 347 | 0.79 | 0.79 | 0.78 | 0.75 | 0.45 | 0.68 |
| Item39 | 347 | 0.59 | 0.58 | 0.56 | 0.53 | 0.60 | 0.70 |
| Item41 | 347 | 0.53 | 0.52 | 0.47 | 0.45 | 0.56 | 0.75 |
| Item47 | 347 | 0.63 | 0.64 | 0.61 | 0.58 | 0.29 | 0.61 |
| Item48 | 347 | 0.71 | 0.70 | 0.68 | 0.66 | 0.76 | 0.80 |

raw.r is the correlation of the item with the entire scale, not correcting for item overlap.

std.r is the correlation of the item with the entire scale, if each item were standardized.

r.drop is the correlation of the item with the scale composed of the remaining items.

Cronbach’s Alpha for Depression dimension

| raw α | std. α | G6(smc) | average r | S/N | ase | mean | sd | median_r |
| --- | --- | --- | --- | --- | --- | --- | --- | --- |
| 0.87 | 0.88 | 0.87 | 0.54 | 7.1 | 0.011 | 0.37 | 0.52 | 0.54 |

- raw α: alpha based upon the covariances
- std. α: The standardized alpha based upon the correlations
- G6(smc): Guttman's Lambda 6 reliability
- smc = (the squared multiple correlation)
- Average r: The average interitem correlation
- S/N: Signal/Noise ratio (the ratio of reliable variance to unreliable variance)
- Ase: alpha standard error
- mean: For data matrices, the mean of the scale formed by summing the items
- sd: For data matrices, the standard deviation of the total score
- r: The correlation of each item with the total score (not corrected for item overlap)
- median-r: The median interitem correlation

| lower95% confidence boundary | alpha | upper 95% confidence boundary |
| --- | --- | --- |
| 0.85 | 0.87 | 0.89 |

Reliability if an item is dropped:

|  | raw_α | std.α | G6(smc) | average r | S/N | alpha se | var.r | med.r |
| --- | --- | --- | --- | --- | --- | --- | --- | --- |
| Item28 | 0.85 | 0.85 | 0.83 | 0.54 | 5.8 | 0.013 | 0.0160 | 0.59 |
| Item30 | 0.85 | 0.85 | 0.84 | 0.54 | 5.8 | 0.013 | 0.0141 | 0.52 |
| Item33 | 0.84 | 0.84 | 0.83 | 0.52 | 5.4 | 0.013 | 0.0118 | 0.53 |
| Item34 | 0.84 | 0.84 | 0.82 | 0.52 | 5.3 | 0.014 | 0.0085 | 0.52 |
| Item35 | 0.85 | 0.85 | 0.84 | 0.54 | 5.9 | 0.013 | 0.0121 | 0.53 |
| Item46 | 0.88 | 0.88 | 0.87 | 0.60 | 7.6 | 0.010 | 0.0040 | 0.62 |

Item statistics

|  | n | raw.r | std.r | r.cor | r.drop | mean | sd |
| --- | --- | --- | --- | --- | --- | --- | --- |
| Item28 | 347 | 0.80 | 0.80 | 0.75 | 0.69 | 0.46 | 0.73 |
| Item30 | 347 | 0.80 | 0.80 | 0.74 | 0.70 | 0.42 | 0.70 |
| Item33 | 347 | 0.82 | 0.83 | 0.80 | 0.74 | 0.21 | 0.59 |
| Item34 | 347 | 0.84 | 0.84 | 0.82 | 0.76 | 0.28 | 0.59 |
| Item35 | 347 | 0.80 | 0.79 | 0.74 | 0.68 | 0.37 | 0.72 |
| Item46 | 347 | 0.66 | 0.66 | 0.55 | 0.51 | 0.46 | 0.67 |

# Cronbach’s Alpha for Anxiety dimension

| raw α | std. α | G6(smc) | average r | S/N | ase | mean | sd | median_r |
| --- | --- | --- | --- | --- | --- | --- | --- | --- |
| 0.89 | 0.89 | 0.90 | 0.39 | 7.8 | 0.0089 | 0.31 | 0.39 | 0.38 |

- raw α: alpha based upon the covariances
- std. α: The standardized alpha based upon the correlations
- G6(smc): Guttman's Lambda 6 reliability
- smc = (the squared multiple correlation)
- Average r: The average interitem correlation
- S/N: Signal/Noise ratio (the ratio of reliable variance to unreliable variance)
- Ase: alpha standard error
- mean: For data matrices, the mean of the scale formed by summing the items
- sd: For data matrices, the standard deviation of the total score
- r: The correlation of each item with the total score (not corrected for item overlap)
- median-r: The median interitem correlation

| lower95% confidence boundary | alpha | upper 95% confidence boundary |
| --- | --- | --- |
| 0.87 | 0.89 | 0.90 |

Reliability if an item is dropped:

|  | raw_α | std.α | G6(smc) | average r | S/N | alpha se | var.r | med.r |
| --- | --- | --- | --- | --- | --- | --- | --- | --- |
| Item18 | 0.87 | 0.87 | 0.88 | 0.38 | 6.8 | 0.0100 | 0.0115 | 0.36 |
| Item21 | 0.87 | 0.87 | 0.88 | 0.38 | 6.8 | 0.0101 | 0.0097 | 0.38 |
| Item23 | 0.88 | 0.88 | 0.89 | 0.40 | 7.4 | 0.0093 | 0.0133 | 0.39 |
| Item24 | 0.88 | 0.88 | 0.89 | 0.40 | 7.3 | 0.0094 | 0.0119 | 0.39 |
| Item27 | 0.87 | 0.87 | 0.88 | 0.38 | 6.6 | 0.0103 | 0.0098 | 0.36 |
| Item40 | 0.88 | 0.88 | 0.89 | 0.41 | 7.7 | 0.0091 | 0.0115 | 0.40 |
| Item42 | 0.88 | 0.88 | 0.89 | 0.40 | 7.4 | 0.0092 | 0.0134 | 0.40 |
| Item43 | 0.88 | 0.88 | 0.89 | 0.40 | 7.5 | 0.0093 | 0.0135 | 0.39 |
| Item44 | 0.88 | 0.88 | 0.88 | 0.39 | 7.0 | 0.0097 | 0.0130 | 0.36 |
| Item45 | 0.87 | 0.87 | 0.88 | 0.38 | 6.9 | 0.0101 | 0.0114 | 0.37 |
| Item49 | 0.88 | 0.88 | 0.89 | 0.40 | 7.2 | 0.0095 | 0.0132 | 0.37 |
| Item50 | 0.88 | 0.88 | 0.89 | 0.40 | 7.3 | 0.0094 | 0.0115 | 0.39 |

Item statistics

|  | n | raw.r | std.r | r.cor | r.drop | mean | sd |
| --- | --- | --- | --- | --- | --- | --- | --- |
| Item18 | 347 | 0.75 | 0.75 | 0.73 | 0.68 | 0.31 | 0.58 |
| Item21 | 347 | 0.76 | 0.75 | 0.75 | 0.69 | 0.31 | 0.58 |
| Item23 | 347 | 0.60 | 0.61 | 0.56 | 0.52 | 0.20 | 0.53 |
| Item24 | 347 | 0.63 | 0.64 | 0.60 | 0.55 | 0.27 | 0.55 |
| Item27 | 347 | 0.80 | 0.79 | 0.79 | 0.74 | 0.31 | 0.59 |
| Item40 | 347 | 0.54 | 0.55 | 0.49 | 0.45 | 0.24 | 0.55 |
| Item42 | 347 | 0.59 | 0.60 | 0.54 | 0.51 | 0.28 | 0.56 |
| Item43 | 347 | 0.57 | 0.60 | 0.54 | 0.51 | 0.13 | 0.40 |
| Item44 | 347 | 0.71 | 0.69 | 0.67 | 0.62 | 0.48 | 0.69 |
| Item45 | 347 | 0.74 | 0.73 | 0.71 | 0.68 | 0.30 | 0.62 |
| Item49 | 347 | 0.66 | 0.65 | 0.60 | 0.57 | 0.43 | 0.66 |
| Item50 | 347 | 0.64 | 0.64 | 0.59 | 0.55 | 0.41 | 0.63 |

# Cronbach’s Alpha for Somatization dimension

| raw α | std. α | G6(smc) | average r | S/N | ase | mean | sd | median_r |
| --- | --- | --- | --- | --- | --- | --- | --- | --- |
| 0.86 | 0.86 | 0.89 | 0.28 | 6.4 | 0.01 | 0.36 | 0.32 | 0.30 |

- raw α: alpha based upon the covariances
- std. α: The standardized alpha based upon the correlations
- G6(smc): Guttman's Lambda 6 reliability
- smc = (the squared multiple correlation)
- Average r: The average interitem correlation
- S/N: Signal/Noise ratio (the ratio of reliable variance to unreliable variance)
- Ase: alpha standard error
- mean: For data matrices, the mean of the scale formed by summing the items
- sd: For data matrices, the standard deviation of the total score
- r: The correlation of each item with the total score (not corrected for item overlap)
- median-r: The median interitem correlation

| lower95% confidence boundary | alpha | upper 95% confidence boundary |
| --- | --- | --- |
| 0.84 | 0.86 | 0.88 |

Reliability if an item is dropped:

|  | raw_α | std.α | G6(smc) | average r | S/N | alpha se | var.r | med.r |
| --- | --- | --- | --- | --- | --- | --- | --- | --- |
| Item1 | 0.86 | 0.86 | 0.88 | 0.28 | 5.9 | 0.011 | 0.014 | 0.29 |
| Item2 | 0.85 | 0.85 | 0.88 | 0.27 | 5.6 | 0.012 | 0.013 | 0.26 |
| Item3 | 0.86 | 0.87 | 0.89 | 0.30 | 6.4 | 0.011 | 0.013 | 0.31 |
| Item4 | 0.86 | 0.86 | 0.88 | 0.29 | 6.1 | 0.011 | 0.013 | 0.31 |
| Item5 | 0.86 | 0.86 | 0.88 | 0.28 | 6.0 | 0.011 | 0.013 | 0.30 |
| Item6 | 0.86 | 0.86 | 0.89 | 0.29 | 6.3 | 0.011 | 0.014 | 0.31 |
| Item7 | 0.86 | 0.86 | 0.89 | 0.28 | 5.9 | 0.011 | 0.015 | 0.30 |
| Item8 | 0.86 | 0.86 | 0.88 | 0.28 | 6.0 | 0.011 | 0.015 | 0.30 |
| Item9 | 0.86 | 0.86 | 0.89 | 0.29 | 6.1 | 0.011 | 0.014 | 0.30 |
| Item10 | 0.86 | 0.86 | 0.89 | 0.29 | 6.1 | 0.011 | 0.015 | 0.31 |
| Item11 | 0.86 | 0.86 | 0.88 | 0.29 | 6.0 | 0.011 | 0.013 | 0.30 |
| Item12 | 0.85 | 0.85 | 0.88 | 0.28 | 5.8 | 0.011 | 0.014 | 0.28 |
| Item13 | 0.85 | 0.85 | 0.88 | 0.28 | 5.8 | 0.011 | 0.014 | 0.30 |
| Item14 | 0.85 | 0.85 | 0.88 | 0.28 | 5.8 | 0.011 | 0.014 | 0.28 |
| Item15 | 0.85 | 0.85 | 0.88 | 0.28 | 5.7 | 0.011 | 0.013 | 0.30 |
| Item16 | 0.86 | 0.85 | 0.88 | 0.28 | 5.9 | 0.011 | 0.014 | 0.30 |

Item statistics

|  | n | raw.r | std.r | r.cor | r.drop | mean | sd |
| --- | --- | --- | --- | --- | --- | --- | --- |
| Item1 | 347 | 0.58 | 0.60 | 0.57 | 0.51 | 0.27 | 0.51 |
| Item2 | 347 | 0.76 | 0.73 | 0.72 | 0.69 | 0.57 | 0.71 |
| Item3 | 347 | 0.34 | 0.40 | 0.34 | 0.29 | 0.04 | 0.25 |
| Item4 | 347 | 0.55 | 0.53 | 0.49 | 0.46 | 0.39 | 0.59 |
| Item5 | 347 | 0.61 | 0.57 | 0.56 | 0.51 | 0.59 | 0.72 |
| Item6 | 347 | 0.45 | 0.46 | 0.40 | 0.37 | 0.25 | 0.51 |
| Item7 | 347 | 0.57 | 0.59 | 0.54 | 0.49 | 0.29 | 0.55 |
| Item8 | 347 | 0.59 | 0.57 | 0.53 | 0.49 | 0.67 | 0.67 |
| Item9 | 347 | 0.53 | 0.51 | 0.46 | 0.43 | 0.78 | 0.67 |
| Item10 | 347 | 0.50 | 0.51 | 0.46 | 0.43 | 0.25 | 0.46 |
| Item11 | 347 | 0.52 | 0.55 | 0.53 | 0.45 | 0.20 | 0.47 |
| Item12 | 347 | 0.63 | 0.63 | 0.61 | 0.56 | 0.35 | 0.57 |
| Item13 | 347 | 0.65 | 0.65 | 0.64 | 0.57 | 0.42 | 0.64 |
| Item14 | 347 | 0.63 | 0.62 | 0.59 | 0.55 | 0.25 | 0.55 |
| Item15 | 347 | 0.63 | 0.66 | 0.65 | 0.56 | 0.24 | 0.54 |
| Item16 | 347 | 0.59 | 0.60 | 0.57 | 0.53 | 0.22 | 0.46 |

# Cronbach’s Alpha for total score

| raw α | std. α | G6(smc) | average r | S/N | ase | mean | sd | median_r |
| --- | --- | --- | --- | --- | --- | --- | --- | --- |
| 0.96 | 0.96 | 0.98 | 0.32 | 23 | 0.003 | 0.40 | 0.36 | 0.31 |

- raw α: alpha based upon the covariances
- std. α: The standardized alpha based upon the correlations
- G6(smc): Guttman's Lambda 6 reliability
- smc = (the squared multiple correlation)
- Average r: The average interitem correlation
- S/N: Signal/Noise ratio (the ratio of reliable variance to unreliable variance)
- Ase: alpha standard error
- mean: For data matrices, the mean of the scale formed by summing the items
- sd: For data matrices, the standard deviation of the total score
- r: The correlation of each item with the total score (not corrected for item overlap)
- median-r: The median interitem correlation

| lower95% confidence boundary | alpha | upper 95% confidence boundary |
| --- | --- | --- |
| 0.95 | 0.96 | 0.96 |

Reliability if an item is dropped:

|  | raw_α | std.α | G6(smc) | average r | S/N | alpha se | var.r | med.r |
| --- | --- | --- | --- | --- | --- | --- | --- | --- |
| Item17 | 0.96 | 0.96 | 0.98 | 0.31 | 22 | 0.0032 | 0.022 | 0.31 |
| Item19 | 0.96 | 0.96 | 0.98 | 0.31 | 22 | 0.0032 | 0.022 | 0.31 |
| Item20 | 0.96 | 0.96 | 0.98 | 0.32 | 23 | 0.0031 | 0.022 | 0.32 |
| Item22 | 0.96 | 0.96 | 0.98 | 0.32 | 23 | 0.0031 | 0.022 | 0.31 |
| Item25 | 0.96 | 0.96 | 0.98 | 0.32 | 23 | 0.0031 | 0.022 | 0.31 |
| Item26 | 0.96 | 0.96 | 0.98 | 0.32 | 23 | 0.0031 | 0.022 | 0.31 |
| Item29 | 0.96 | 0.96 | 0.98 | 0.31 | 22 | 0.0032 | 0.021 | 0.31 |
| Item31 | 0.96 | 0.96 | 0.98 | 0.31 | 22 | 0.0032 | 0.021 | 0.31 |
| Item32 | 0.96 | 0.96 | 0.98 | 0.31 | 22 | 0.0032 | 0.021 | 0.31 |
| Item36 | 0.96 | 0.96 | 0.98 | 0.31 | 22 | 0.0032 | 0.021 | 0.31 |
| Item37 | 0.96 | 0.96 | 0.98 | 0.31 | 22 | 0.0032 | 0.022 | 0.31 |
| Item38 | 0.96 | 0.96 | 0.98 | 0.31 | 22 | 0.0032 | 0.022 | 0.31 |
| Item39 | 0.96 | 0.96 | 0.98 | 0.32 | 23 | 0.0031 | 0.022 | 0.31 |
| Item41 | 0.96 | 0.96 | 0.98 | 0.32 | 23 | 0.0030 | 0.021 | 0.32 |
| Item47 | 0.96 | 0.96 | 0.98 | 0.32 | 23 | 0.0031 | 0.022 | 0.31 |
| Item48 | 0.96 | 0.96 | 0.98 | 0.32 | 23 | 0.0032 | 0.022 | 0.31 |
| Item28 | 0.96 | 0.96 | 0.98 | 0.31 | 22 | 0.0032 | 0.022 | 0.31 |
| Item30 | 0.96 | 0.96 | 0.98 | 0.31 | 22 | 0.0032 | 0.021 | 0.31 |
| Item33 | 0.96 | 0.96 | 0.98 | 0.32 | 23 | 0.0031 | 0.021 | 0.31 |
| Item34 | 0.96 | 0.96 | 0.98 | 0.31 | 22 | 0.0032 | 0.021 | 0.31 |
| Item35 | 0.96 | 0.96 | 0.98 | 0.32 | 23 | 0.0031 | 0.021 | 0.31 |
| Item46 | 0.96 | 0.96 | 0.98 | 0.32 | 23 | 0.0031 | 0.022 | 0.31 |
| Item18 | 0.96 | 0.96 | 0.98 | 0.32 | 23 | 0.0031 | 0.022 | 0.31 |
| Item21 | 0.96 | 0.96 | 0.98 | 0.32 | 23 | 0.0031 | 0.022 | 0.31 |
| Item23 | 0.96 | 0.96 | 0.98 | 0.32 | 23 | 0.0031 | 0.022 | 0.31 |
| Item24 | 0.96 | 0.96 | 0.98 | 0.32 | 23 | 0.0031 | 0.022 | 0.31 |
| Item27 | 0.96 | 0.96 | 0.98 | 0.31 | 23 | 0.0031 | 0.022 | 0.31 |
| Item40 | 0.96 | 0.96 | 0.98 | 0.32 | 23 | 0.0031 | 0.022 | 0.31 |
| Item42 | 0.96 | 0.96 | 0.98 | 0.32 | 23 | 0.0031 | 0.022 | 0.31 |
| Item43 | 0.96 | 0.96 | 0.98 | 0.32 | 23 | 0.0031 | 0.022 | 0.31 |
| Item44 | 0.96 | 0.96 | 0.98 | 0.32 | 23 | 0.0031 | 0.022 | 0.31 |
| Item45 | 0.96 | 0.96 | 0.98 | 0.32 | 23 | 0.0031 | 0.022 | 0.31 |
| Item49 | 0.96 | 0.96 | 0.98 | 0.32 | 23 | 0.0031 | 0.022 | 0.31 |
| Item50 | 0.96 | 0.96 | 0.98 | 0.32 | 23 | 0.0031 | 0.022 | 0.31 |
| Item1 | 0.96 | 0.96 | 0.98 | 0.32 | 23 | 0.0031 | 0.022 | 0.31 |
| Item2 | 0.96 | 0.96 | 0.98 | 0.32 | 23 | 0.0031 | 0.022 | 0.31 |
| Item3 | 0.96 | 0.96 | 0.98 | 0.32 | 24 | 0.0031 | 0.021 | 0.32 |
| Item4 | 0.96 | 0.96 | 0.98 | 0.32 | 24 | 0.0030 | 0.021 | 0.32 |
| Item5 | 0.96 | 0.96 | 0.98 | 0.32 | 23 | 0.0030 | 0.021 | 0.32 |
| Item6 | 0.96 | 0.96 | 0.98 | 0.32 | 23 | 0.0031 | 0.022 | 0.32 |
| Item7 | 0.96 | 0.96 | 0.98 | 0.32 | 23 | 0.0031 | 0.023 | 0.31 |
| Item8 | 0.96 | 0.96 | 0.98 | 0.32 | 23 | 0.0031 | 0.023 | 0.32 |
| Item9 | 0.96 | 0.96 | 0.98 | 0.32 | 23 | 0.0030 | 0.022 | 0.32 |
| Item10 | 0.96 | 0.96 | 0.98 | 0.32 | 23 | 0.0031 | 0.022 | 0.32 |
| Item11 | 0.96 | 0.96 | 0.98 | 0.32 | 23 | 0.0031 | 0.022 | 0.32 |
| Item12 | 0.96 | 0.96 | 0.98 | 0.32 | 23 | 0.0031 | 0.022 | 0.31 |
| Item13 | 0.96 | 0.96 | 0.98 | 0.32 | 23 | 0.0031 | 0.022 | 0.31 |
| Item14 | 0.96 | 0.96 | 0.98 | 0.32 | 23 | 0.0031 | 0.022 | 0.32 |
| Item15 | 0.96 | 0.96 | 0.98 | 0.32 | 23 | 0.0031 | 0.022 | 0.31 |
| Item16 | 0.96 | 0.96 | 0.98 | 0.32 | 23 | 0.0031 | 0.023 | 0.31 |

Item statistics

|  | n | raw.r | std.r | r.cor | r.drop | mean | sd |
| --- | --- | --- | --- | --- | --- | --- | --- |
| Item17 | 347 | 0.70 | 0.69 | 0.69 | 0.68 | 0.58 | 0.69 |
| Item19 | 347 | 0.74 | 0.73 | 0.73 | 0.72 | 0.62 | 0.67 |
| Item20 | 347 | 0.50 | 0.49 | 0.49 | 0.47 | 0.70 | 0.75 |
| Item22 | 347 | 0.68 | 0.67 | 0.66 | 0.66 | 0.90 | 0.72 |
| Item25 | 347 | 0.66 | 0.66 | 0.66 | 0.64 | 0.44 | 0.64 |
| Item26 | 347 | 0.65 | 0.64 | 0.63 | 0.62 | 0.80 | 0.76 |
| Item29 | 347 | 0.70 | 0.69 | 0.69 | 0.68 | 0.33 | 0.67 |
| Item31 | 347 | 0.75 | 0.74 | 0.74 | 0.73 | 0.36 | 0.68 |
| Item32 | 347 | 0.73 | 0.73 | 0.72 | 0.71 | 0.36 | 0.66 |
| Item36 | 347 | 0.74 | 0.74 | 0.74 | 0.73 | 0.27 | 0.60 |
| Item37 | 347 | 0.75 | 0.74 | 0.74 | 0.73 | 0.51 | 0.74 |
| Item38 | 347 | 0.76 | 0.76 | 0.75 | 0.75 | 0.45 | 0.68 |
| Item39 | 347 | 0.53 | 0.51 | 0.51 | 0.50 | 0.60 | 0.70 |
| Item41 | 347 | 0.42 | 0.39 | 0.38 | 0.38 | 0.56 | 0.75 |
| Item47 | 347 | 0.64 | 0.64 | 0.64 | 0.62 | 0.29 | 0.61 |
| Item48 | 347 | 0.69 | 0.67 | 0.67 | 0.66 | 0.76 | 0.80 |
| Item28 | 347 | 0.72 | 0.72 | 0.72 | 0.70 | 0.46 | 0.73 |
| Item30 | 347 | 0.71 | 0.70 | 0.70 | 0.69 | 0.42 | 0.70 |
| Item33 | 347 | 0.66 | 0.66 | 0.66 | 0.64 | 0.21 | 0.59 |
| Item34 | 347 | 0.75 | 0.74 | 0.74 | 0.73 | 0.28 | 0.59 |
| Item35 | 347 | 0.59 | 0.57 | 0.57 | 0.56 | 0.37 | 0.72 |
| Item46 | 347 | 0.62 | 0.62 | 0.61 | 0.60 | 0.46 | 0.67 |
| Item18 | 347 | 0.64 | 0.65 | 0.64 | 0.62 | 0.31 | 0.58 |
| Item21 | 347 | 0.64 | 0.64 | 0.64 | 0.62 | 0.31 | 0.58 |
| Item23 | 347 | 0.57 | 0.58 | 0.57 | 0.55 | 0.20 | 0.53 |
| Item24 | 347 | 0.55 | 0.56 | 0.55 | 0.52 | 0.27 | 0.55 |
| Item27 | 347 | 0.68 | 0.69 | 0.68 | 0.67 | 0.31 | 0.59 |
| Item40 | 347 | 0.53 | 0.53 | 0.52 | 0.51 | 0.24 | 0.55 |
| Item42 | 347 | 0.54 | 0.54 | 0.53 | 0.51 | 0.28 | 0.56 |
| Item43 | 347 | 0.53 | 0.55 | 0.54 | 0.51 | 0.13 | 0.40 |
| Item44 | 347 | 0.63 | 0.62 | 0.62 | 0.60 | 0.48 | 0.69 |
| Item45 | 347 | 0.66 | 0.65 | 0.65 | 0.64 | 0.30 | 0.62 |
| Item49 | 347 | 0.50 | 0.50 | 0.49 | 0.47 | 0.43 | 0.66 |
| Item50 | 347 | 0.53 | 0.54 | 0.53 | 0.51 | 0.41 | 0.63 |
| Item1 | 347 | 0.50 | 0.51 | 0.50 | 0.48 | 0.27 | 0.51 |
| Item2 | 347 | 0.52 | 0.52 | 0.51 | 0.49 | 0.57 | 0.71 |
| Item3 | 347 | 0.26 | 0.28 | 0.26 | 0.24 | 0.04 | 0.25 |
| Item4 | 347 | 0.27 | 0.27 | 0.26 | 0.24 | 0.39 | 0.59 |
| Item5 | 347 | 0.36 | 0.36 | 0.35 | 0.33 | 0.59 | 0.72 |
| Item6 | 347 | 0.37 | 0.38 | 0.37 | 0.34 | 0.25 | 0.51 |
| Item7 | 347 | 0.52 | 0.54 | 0.52 | 0.50 | 0.29 | 0.55 |
| Item8 | 347 | 0.48 | 0.48 | 0.47 | 0.45 | 0.67 | 0.67 |
| Item9 | 347 | 0.35 | 0.36 | 0.34 | 0.32 | 0.78 | 0.67 |
| Item10 | 347 | 0.42 | 0.43 | 0.42 | 0.40 | 0.25 | 0.46 |
| Item11 | 347 | 0.49 | 0.51 | 0.50 | 0.47 | 0.20 | 0.47 |
| Item12 | 347 | 0.49 | 0.50 | 0.49 | 0.46 | 0.35 | 0.57 |
| Item13 | 347 | 0.50 | 0.51 | 0.50 | 0.47 | 0.42 | 0.64 |
| Item14 | 347 | 0.46 | 0.48 | 0.47 | 0.44 | 0.25 | 0.55 |
| Item15 | 347 | 0.54 | 0.56 | 0.55 | 0.52 | 0.24 | 0.54 |
| Item16 | 347 | 0.54 | 0.55 | 0.54 | 0.52 | 0.22 | 0.46 |
