# Supplementary material for: Fear at the time of the COVID-19 pandemic: validation of the Arabic version of the Four-Dimensional Symptom Questionnaire among Saudi-based respondents
Source: BJPsych Open. 2021 Jan 12;7(1):e33. doi: 10.1192/bjo.2020.166 (PMC7804080; doi:10.1192/bjo.2020.166)
Supplement: Supplementary file 1 [file S2056472420001660sup.zip › S2056472420001660sup003.docx]

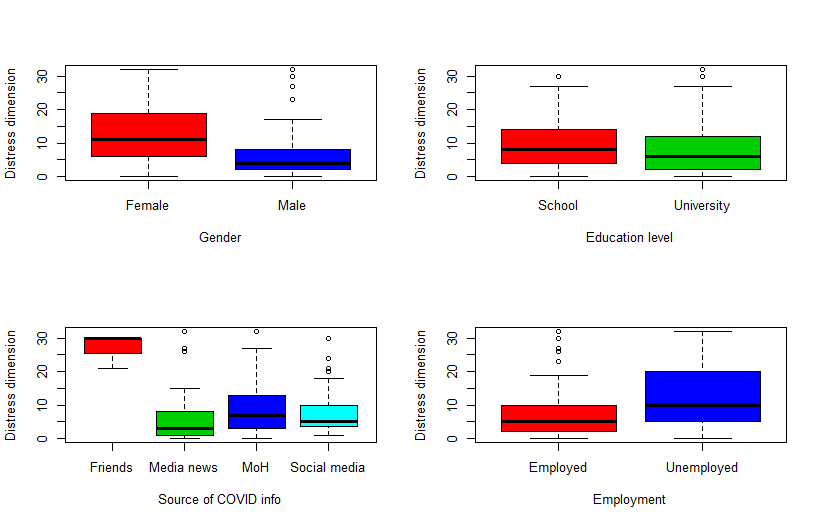


**Figure 1: Effects of background factors on distress score**

**Table 1. Estimates of the coefficients for background factors on distress dimension score**

|  | Estimate | Odds | odds’ 95% CI | SE | t value | P value |
| --- | --- | --- | --- | --- | --- | --- |
| **Separated** | 0.378 | 1.459 | 1.148 to 1.830 | 0.119 | 3.186 | 0.001 *** |
| **Single** | 0.108 | 1.114 | 0.998 to 1.244 | 0.056 | 1.925 | 0.054 . |
| **Widowed** | 0.461 | 1.586 | 1.101 to 2.213 | 0.178 | 2.595 | 0.009 ** |
| **Age** | -0.031 | 0.969 | 0.965 to 0.975 | 0.003 | -11.322 | <0.001*** |
| **Male Sex** | -0.355 | 0.701 | 0.639 to 0.769 | 0.047 | -7.563 | <0.001*** |
| **University Edu** | -0.177 | 0.838 | 0.759 to 0.925 | 0.050 | -3.531 | <0.001*** |
| **Friends’ Info** | 0.743 | 2.102 | 1.772 to 2.483 | 0.086 | 8.643 | <0.001*** |
| **Media news** | -0.288 | 0.750 | 0.666 to 0.841 | 0.060 | -4.842 | <0.001*** |
| **Social media** | -0.175 | 0.839 | 0.756 to 0.931 | 0.053 | -3.289 | 0.001 *** |
| **Unemployment** | 0.032 | 1.033 | 0.932 to 1.143 | 0.052 | 0.611 | 0.541 |


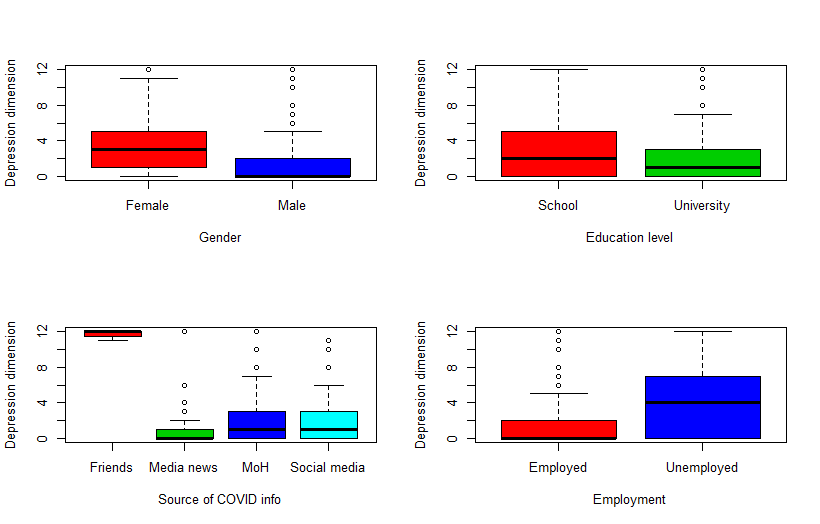


**Figure 2: Effects of background factors on depression score**

**Table 2. Estimates of the coefficients for background factors on depression dimension score**

|  | Estimate | Odds | odds’ 95% CI | SE | t value | P value |
| --- | --- | --- | --- | --- | --- | --- |
| **Separated** | 0.081 | 1.084 | 0.661 to 1.683 | 0.237 | 0.343 | 0.731 |
| **Single** | 0.107 | 1.113 | 0.887 to 1.398 | 0.116 | 0.925 | 0.355 |
| **Widowed** | 0.505 | 1.657 | 0.857 to 2.918 | 0.310 | 1.629 | 0.103 |
| **Age** | -0.017 | 0.983 | 0.974 to 0.993 | 0.005 | -3.424 | 0.001 *** |
| **Male Sex** | -0.605 | 0.546 | 0.455 to 0.655 | 0.093 | -6.518 | <0.001*** |
| **University Edu** | -0.351 | 0.704 | 0.589 to 0.843 | 0.091 | -3.838 | <0.001*** |
| **Friends’ Info** | 1.166 | 3.209 | 2.421 to 4.214 | 0.141 | 8.253 | <0.001*** |
| **Media news** | -0.735 | 0.480 | 0.358 to 0.631 | 0.144 | -5.085 | <0.001*** |
| **Social media** | -0.104 | 0.901 | 0.737 to 1.096 | 0.101 | -1.029 | 0.303 |
| **Unemployment** | 0.343 | 1.409 | 1.153 to 1.723 | 0.103 | 3.344 | 0.001 *** |


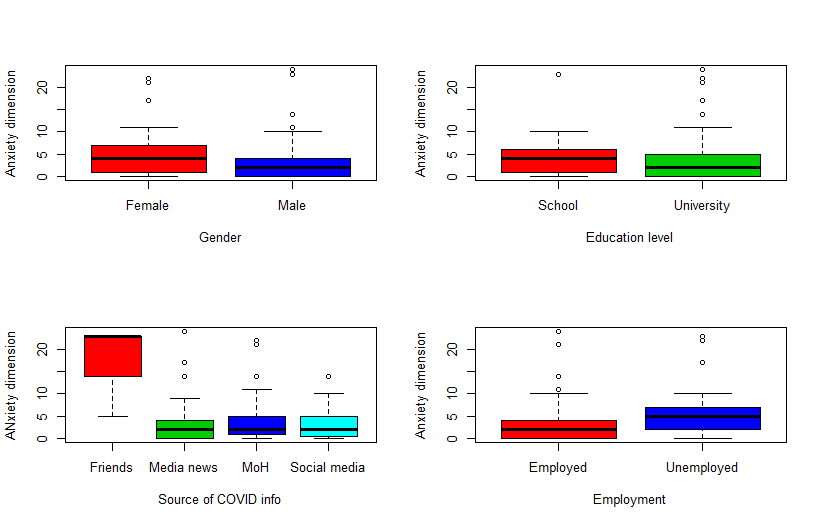


**Figure 3: Effects of background factors on anxiety score**

**Table 3. Estimates of the coefficients for background factors on anxiety dimension score**

|  | Estimate | Odds | odds’ 95% CI | SE | t value | P value |
| --- | --- | --- | --- | --- | --- | --- |
| **Separated** | 0.246 | 1.279 | 0.864 to 1.827 | 0.190 | 1.294 | 0.196 |
| **Single** | -0.136 | 0.873 | 0.732 to 1.040 | 0.089 | -1.524 | 0.128 |
| **Widowed** | 0.525 | 1.690 | 0.965 to 2.763 | 0.267 | 1.970 | 0.049 * |
| **Age** | -0.033 | 0.968 | 0.960 to 0.976 | 0.004 | -7.899 | <0.001*** |
| **Male Sex** | -0.178 | 0.837 | 0.727 to 0.964 | 0.072 | -2.471 | 0.013 * |
| **University Edu** | -0.231 | 0.794 | 0.685 to 0.921 | 0.076 | -3.057 | 0.002 ** |
| **Friends’ Info** | 1.212 | 3.360 | 2.664 to 4.215 | 0.117 | 10.365 | <0.001*** |
| **Media news** | -0.143 | 0.867 | 0.729 to 1.024 | 0.087 | -1.653 | 0.098 . |
| **Social media** | -0.122 | 0.885 | 0.753 to 1.036 | 0.081 | -1.500 | 0.133 |
| **Unemployment** | 0.133 | 1.142 | 0.970 to 1.346 | 0.083 | 1.600 | 0.110 |


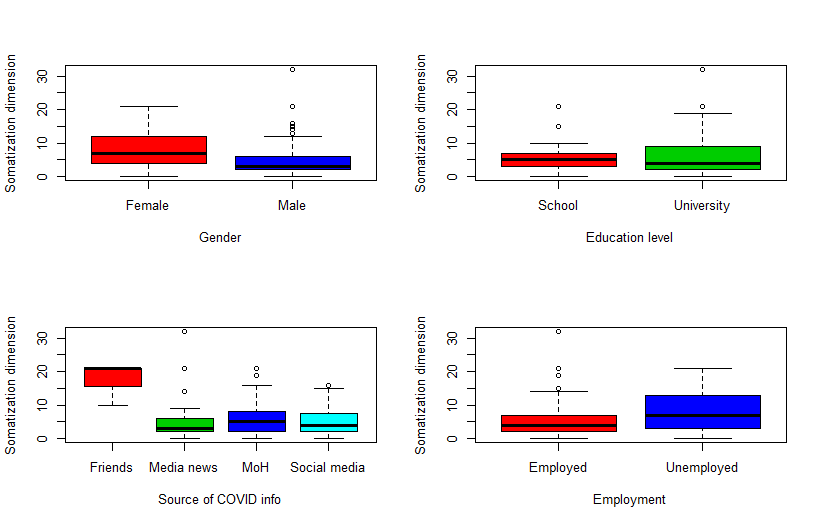


**Figure 4: Effects of background factors on somatization score**

**Table 4. Estimates of the coefficients for background factors on somatization dimension score**

|  | Estimate | Odds | odds’ 95% CI | SE | t value | P value |
| --- | --- | --- | --- | --- | --- | --- |
| **Separated** | -0.169 | 0.845 | 0.590 to 1.171 | 0.174 | -0.967 | 0.333 |
| **Single** | 0.155 | 1.168 | 1.021 to 1.336 | 0.068 | 2.268 | 0.023 * |
| **Widowed** | -0.091 | 0.913 | 0.507 to 1.507 | 0.276 | -0.330 | 0.741 |
| **Age** | -0.017 | 0.983 | 0.977 to 0.990 | 0.003 | -5.140 | <0.001*** |
| **Male Sex** | -0.351 | 0.704 | 0.630 to 0.787 | 0.057 | -6.182 | <0.001*** |
| **University Edu** | 0.143 | 1.154 | 1.018 to 1.311 | 0.064 | 2.220 | 0.026 * |
| **Friends’ Info** | 0.883 | 2.418 | 1.958 to 2.967 | 0.106 | 8.331 | <0.001*** |
| **Media news** | -0.046 | 0.955 | 0.839 to 1.084 | 0.065 | -0.703 | 0.482 |
| **Social media** | -0.092 | 0.912 | 0.801 to 1.036 | 0.066 | -1.398 | 0.162 |
| **Unemployment** | 0.154 | 1.166 | 1.031 to 1.321 | 0.063 | 2.439 | 0.015 * |

**Figure 5: Effects of background factors on total 4DSQ score**


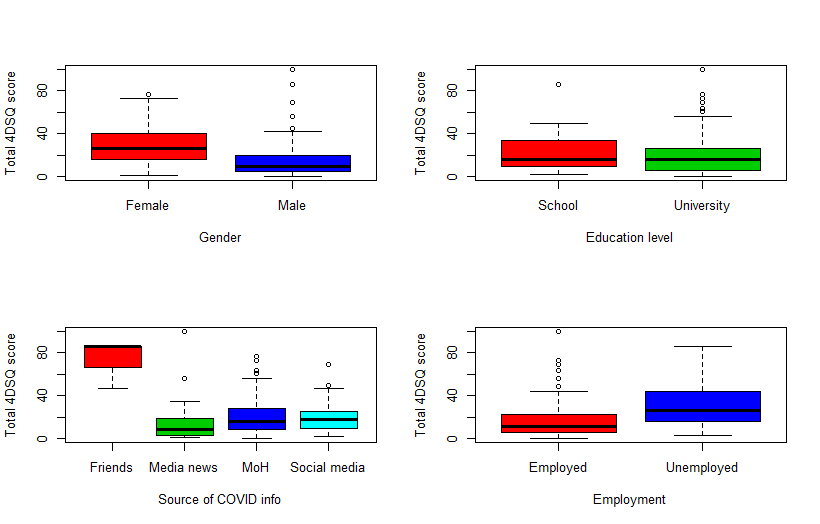


**Table 5. Estimates of the coefficients for background factors on total 4DSQ score**

|  | Estimate | Odds | odds’ 95% CI | SE | t value | P value |
| --- | --- | --- | --- | --- | --- | --- |
| **Separated** | 0.177 | 1.194 | 1.013 to 1.396 | 0.082 | 2.165 | 0.030 * |
| **Single** | 0.079 | 1.082 | 1.006 to 1.163 | 0.037 | 2.124 | 0.034 * |
| **Widowed** | 0.348 | 1.416 | 1.112 to 1.778 | 0.120 | 2.909 | 0.004 ** |
| **Age** | -0.025 | 0.975 | 0.972 to 0.978 | 0.002 | -14.608 | <0.001*** |
| **Male Sex** | -0.350 | 0.705 | 0.664 to 0.748 | 0.030 | -11.495 | <0.001*** |
| **University Edu** | -0.125 | 0.882 | 0.828 to 0.941 | 0.033 | -3.844 | <0.001*** |
| **Friends’ Info** | 0.930 | 2.535 | 2.281 to 2.811 | 0.053 | 17.451 | <0.001*** |
| **Media news** | -0.223 | 0.800 | 0.743 to 0.861 | 0.038 | -5.901 | <0.001*** |
| **Social media** | -0.135 | 0.874 | 0.816 to 0.935 | 0.035 | -3.898 | <0.001*** |
| **Unemployment** | 0.120 | 1.127 | 1.054 to 1.205 | 0.034 | 3.517 | <0.001*** |

**Table 4. Estimates of the coefficients for background factors on 4DSQ score and dimensions**

|  | Estimate | Odds | odds’ 95% CI | SE | t value | P value |
| --- | --- | --- | --- | --- | --- | --- |
| **Effect on Total 4SDQ score** | | | | | | |
| **Separated** | 0.177 | 1.194 | 1.013 to 1.396 | 0.082 | 2.165 | 0.030 * |
| **Single** | 0.079 | 1.082 | 1.006 to 1.163 | 0.037 | 2.124 | 0.034 * |
| **Widowed** | 0.348 | 1.416 | 1.112 to 1.778 | 0.120 | 2.909 | 0.004 ** |
| **Age** | -0.025 | 0.975 | 0.972 to 0.978 | 0.002 | -14.608 | <0.001*** |
| **Male Sex** | -0.350 | 0.705 | 0.664 to 0.748 | 0.030 | -11.495 | <0.001*** |
| **University Edu** | -0.125 | 0.882 | 0.828 to 0.941 | 0.033 | -3.844 | <0.001*** |
| **Friends’ Info** | 0.930 | 2.535 | 2.281 to 2.811 | 0.053 | 17.451 | <0.001*** |
| **Media news** | -0.223 | 0.800 | 0.743 to 0.861 | 0.038 | -5.901 | <0.001*** |
| **Social media** | -0.135 | 0.874 | 0.816 to 0.935 | 0.035 | -3.898 | <0.001*** |
| **Unemployment** | 0.120 | 1.127 | 1.054 to 1.205 | 0.034 | 3.517 | <0.001*** |
| **Effect on distress dimension score** | | | | | | |
| **Separated** | 0.378 | 1.459 | 1.148 to 1.830 | 0.119 | 3.186 | 0.001 *** |
| **Single** | 0.108 | 1.114 | 0.998 to 1.244 | 0.056 | 1.925 | 0.054 . |
| **Widowed** | 0.461 | 1.586 | 1.101 to 2.213 | 0.178 | 2.595 | 0.009 ** |
| **Age** | -0.031 | 0.969 | 0.965 to 0.975 | 0.003 | -11.322 | <0.001*** |
| **Male Sex** | -0.355 | 0.701 | 0.639 to 0.769 | 0.047 | -7.563 | <0.001*** |
| **University Edu** | -0.177 | 0.838 | 0.759 to 0.925 | 0.050 | -3.531 | <0.001*** |
| **Friends’ Info** | 0.743 | 2.102 | 1.772 to 2.483 | 0.086 | 8.643 | <0.001*** |
| **Media news** | -0.288 | 0.750 | 0.666 to 0.841 | 0.060 | -4.842 | <0.001*** |
| **Social media** | -0.175 | 0.839 | 0.756 to 0.931 | 0.053 | -3.289 | 0.001 *** |
| **Unemployment** | 0.032 | 1.033 | 0.932 to 1.143 | 0.052 | 0.611 | 0.541 |
| **Effect on depression dimension score** | | | | | | |
| **Separated** | 0.081 | 1.084 | 0.661 to 1.683 | 0.237 | 0.343 | 0.731 |
| **Single** | 0.107 | 1.113 | 0.887 to 1.398 | 0.116 | 0.925 | 0.355 |
| **Widowed** | 0.505 | 1.657 | 0.857 to 2.918 | 0.310 | 1.629 | 0.103 |
| **Age** | -0.017 | 0.983 | 0.974 to 0.993 | 0.005 | -3.424 | 0.001 *** |
| **Male Sex** | -0.605 | 0.546 | 0.455 to 0.655 | 0.093 | -6.518 | <0.001*** |
| **University Edu** | -0.351 | 0.704 | 0.589 to 0.843 | 0.091 | -3.838 | <0.001*** |
| **Friends’ Info** | 1.166 | 3.209 | 2.421 to 4.214 | 0.141 | 8.253 | <0.001*** |
| **Media news** | -0.735 | 0.480 | 0.358 to 0.631 | 0.144 | -5.085 | <0.001*** |
| **Social media** | -0.104 | 0.901 | 0.737 to 1.096 | 0.101 | -1.029 | 0.303 |
| **Unemployment** | 0.343 | 1.409 | 1.153 to 1.723 | 0.103 | 3.344 | 0.001 *** |
| **Effect on anxiety dimension score** | | | | | | |
| **Separated** | 0.246 | 1.279 | 0.864 to 1.827 | 0.190 | 1.294 | 0.196 |
| **Single** | -0.136 | 0.873 | 0.732 to 1.040 | 0.089 | -1.524 | 0.128 |
| **Widowed** | 0.525 | 1.690 | 0.965 to 2.763 | 0.267 | 1.970 | 0.049 * |
| **Age** | -0.033 | 0.968 | 0.960 to 0.976 | 0.004 | -7.899 | <0.001*** |
| **Male Sex** | -0.178 | 0.837 | 0.727 to 0.964 | 0.072 | -2.471 | 0.013 * |
| **University Edu** | -0.231 | 0.794 | 0.685 to 0.921 | 0.076 | -3.057 | 0.002 ** |
| **Friends’ Info** | 1.212 | 3.360 | 2.664 to 4.215 | 0.117 | 10.365 | <0.001*** |
| **Media news** | -0.143 | 0.867 | 0.729 to 1.024 | 0.087 | -1.653 | 0.098 . |
| **Social media** | -0.122 | 0.885 | 0.753 to 1.036 | 0.081 | -1.500 | 0.133 |
| **Unemployment** | 0.133 | 1.142 | 0.970 to 1.346 | 0.083 | 1.600 | 0.110 |
| **Effect on somatization dimension score** | | | | | | |
| **Separated** | -0.169 | 0.845 | 0.590 to 1.171 | 0.174 | -0.967 | 0.333 |
| **Single** | 0.155 | 1.168 | 1.021 to 1.336 | 0.068 | 2.268 | 0.023 * |
| **Widowed** | -0.091 | 0.913 | 0.507 to 1.507 | 0.276 | -0.330 | 0.741 |
| **Age** | -0.017 | 0.983 | 0.977 to 0.990 | 0.003 | -5.140 | <0.001*** |
| **Male Sex** | -0.351 | 0.704 | 0.630 to 0.787 | 0.057 | -6.182 | <0.001*** |
| **University Edu** | 0.143 | 1.154 | 1.018 to 1.311 | 0.064 | 2.220 | 0.026 * |
| **Friends’ Info** | 0.883 | 2.418 | 1.958 to 2.967 | 0.106 | 8.331 | <0.001*** |
| **Media news** | -0.046 | 0.955 | 0.839 to 1.084 | 0.065 | -0.703 | 0.482 |
| **Social media** | -0.092 | 0.912 | 0.801 to 1.036 | 0.066 | -1.398 | 0.162 |
| **Unemployment** | 0.154 | 1.166 | 1.031 to 1.321 | 0.063 | 2.439 | 0.015 * |
