# Supplementary material for: Fear at the time of the COVID-19 pandemic: validation of the Arabic version of the Four-Dimensional Symptom Questionnaire among Saudi-based respondents
Source: BJPsych Open. 2021 Jan 12;7(1):e33. doi: 10.1192/bjo.2020.166 (PMC7804080; doi:10.1192/bjo.2020.166)
Supplement: Supplementary file 1 [file S2056472420001660sup.zip › S2056472420001660sup004.docx]

Results for Exploratory Factor Analysis for 5 factors (Varimax rotation)

Uniquenesses:

| Item28 | Item30 | Item33 | Item34 | Item35 | Item46 | Item18 | Item21 | Item23 |
| --- | --- | --- | --- | --- | --- | --- | --- | --- |
| 0.434 | 0.337 | 0.224 | 0.295 | 0.368 | 0.436 | 0.437 | 0.342 | 0.549 |
| Item24 | Item27 | Item40 | Item42 | Item43 | Item44 | Item45 | Item49 | Item50 |
| 0.568 | 0.265 | 0.616 | 0.678 | 0.534 | 0.490 | 0.421 | 0.716 | 0.522 |
| Item1 | Item2 | Item3 | Item4 | Item5 | Item6 | Item7 | Item8 | Item9 |
| 0.494 | 0.345 | 0.820 | 0.711 | 0.609 | 0.746 | 0.683 | 0.707 | 0.724 |
| Item10 | Item11 | Item12 | Item13 | Item14 | Item15 | Item16 | Item17 | Item19 |
| 0.770 | 0.640 | 0.632 | 0.612 | 0.500 | 0.612 | 0.616 | 0.428 | 0.354 |
| Item20 | Item22 | Item25 | Item26 | Item29 | Item31 | Item32 | Item36 | Item37 |
| 0.734 | 0.456 | 0.475 | 0.452 | 0.327 | 0.228 | 0.319 | 0.188 | 0.346 |
| Item38 | Item39 | Item41 | Item47 | Item48 |  |  |  |  |
| 0.380 | 0.716 | 0.684 | 0.373 | 0.473 |  |  |  |  |

Loadings:

|  | Factor1 | Factor2 | Factor3 | Factor4 | Factor5 |
| --- | --- | --- | --- | --- | --- |
| Item28 | 0.580 | 0.260 | 0.224 | 0.252 | 0.219 |
| Item30 | 0.753 |  | 0.214 |  |  |
| Item33 | 0.775 |  |  |  | 0.365 |
| Item34 | 0.766 |  | 0.270 |  |  |
| Item35 | 0.751 |  |  |  | 0.204 |
| Item40 | 0.583 |  |  |  |  |
| Item44 | 0.556 |  | 0.323 |  | 0.293 |
| Item29 | 0.762 |  |  | 0.224 |  |
| Item31 | 0.803 | 0.236 |  |  |  |
| Item32 | 0.727 | 0.246 | 0.215 |  |  |
| Item36 | 0.858 |  | 0.225 |  |  |
| Item37 | 0.705 | 0.310 |  | 0.209 |  |
| Item38 | 0.622 | 0.305 | 0.239 | 0.278 |  |
| Item47 | 0.610 |  | 0.331 |  | 0.377 |
| Item2 |  | 0.773 |  |  |  |
| Item4 |  | 0.530 |  |  |  |
| Item5 |  | 0.578 |  | 0.230 |  |
| Item13 |  | 0.546 |  |  |  |
| Item14 |  | 0.625 | 0.292 |  |  |
| Item18 | 0.262 | 0.239 | 0.635 |  |  |
| Item21 | 0.316 |  | 0.707 | 0.219 |  |
| Item24 | 0.283 |  | 0.568 |  |  |
| Item27 | 0.366 |  | 0.711 | 0.237 |  |
| Item45 | 0.439 |  | 0.571 |  |  |
| Item50 |  | 0.329 | 0.586 |  |  |
| Item25 | 0.287 | 0.204 | 0.534 | 0.318 |  |
| Item26 | 0.291 | 0.318 | 0.215 | 0.532 |  |
| Item46 | 0.258 | 0.229 | 0.309 | 0.287 | 0.516 |
| Item23 | 0.464 | 0.244 | 0.394 |  |  |
| Item42 | 0.460 |  | 0.306 |  |  |
| Item43 | 0.262 | 0.488 | 0.324 |  |  |
| Item49 | 0.289 |  | 0.416 |  |  |
| Item1 | 0.251 | 0.484 |  |  | 0.419 |
| Item3 |  | 0.240 |  |  | 0.336 |
| Item6 |  | 0.304 | 0.383 |  |  |
| Item7 | 0.232 | 0.386 | 0.261 |  | 0.208 |
| Item8 | 0.201 | 0.457 |  |  |  |
| Item9 |  | 0.384 | 0.276 |  |  |
| Item10 | 0.261 | 0.395 |  |  |  |
| Item11 | 0.203 | 0.297 | 0.368 |  | 0.295 |
| Item12 |  | 0.495 | 0.206 |  | 0.238 |
| Item15 | 0.216 | 0.459 | 0.288 |  | 0.218 |
| Item16 | 0.254 | 0.455 | 0.315 |  |  |
| Item17 | 0.491 | 0.239 | 0.216 | 0.429 | 0.208 |
| Item19 | 0.333 | 0.373 | 0.371 | 0.478 |  |
| Item20 | 0.210 | 0.283 |  | 0.309 |  |
| Item22 | 0.433 | 0.415 |  | 0.406 |  |
| Item39 | 0.261 | 0.284 | 0.210 | 0.301 |  |
| Item41 | 0.436 |  |  | 0.338 |  |
| Item48 | 0.388 | 0.237 | 0.282 | 0.387 | 0.303 |

|  | Factor1 | Factor2 | Factor3 | Factor4 | Factor5 |
| --- | --- | --- | --- | --- | --- |
| SS loadings | 9.850 | 5.475 | 5.206 | 2.325 | 1.759 |
| Proportion Variance | 0.197 | 0.109 | 0.104 | 0.046 | 0.035 |
| Cumulative Variance | 0.197 | 0.306 | 0.411 | 0.457 | 0.492 |

Test of the hypothesis that 5 factors are sufficient.

The chi square statistic is 4211.06 on 985 degrees of freedom.

The p-value is < 0.001
